# Gut lumen-leaked microbial DNA causes myocardial inflammation and impairs cardiac contractility in ageing mouse heart

**DOI:** 10.3389/fimmu.2023.1216344

**Published:** 2023-07-13

**Authors:** Hong Gao, Ke Wang, Jorge A. Suarez, Zhongmou Jin, Karina Cunha e Rocha, Dinghong Zhang, Andrea Farrell, Tyler Truong, Yasemin Tekin, Breanna Tan, Hyun Suh Jung, Julia Kempf, Sushil K. Mahata, Wolfgang H. Dillmann, Jorge Suarez, Wei Ying

**Affiliations:** ^1^ Division of Endocrinology and Metabolism, Department of Medicine, University of California San Diego, San Diego, CA, United States; ^2^ Division of Biological Sciences, University of California San Diego, San Diego, CA, United States; ^3^ the Veterans Affairs San Diego Healthcare System, San Diego, CA, United States; ^4^ Division of Nephrology and Hypertension, Department of Medicine, University of California San Diego, San Diego, CA, United States

**Keywords:** microbial DNA, extracellular vesicle, Vsig4 + macrophage, inflammageing, cardiac contractility

## Abstract

Emerging evidence indicates the critical roles of microbiota in mediating host cardiac functions in ageing, however, the mechanisms underlying the communications between microbiota and cardiac cells during the ageing process have not been fully elucidated. Bacterial DNA was enriched in the cardiomyocytes of both ageing humans and mice. Antibiotic treatment remarkably reduced bacterial DNA abundance in ageing mice. Gut microbial DNA containing extracellular vesicles (mEVs) were readily leaked into the bloodstream and infiltrated into cardiomyocytes in ageing mice, causing cardiac microbial DNA enrichment. Vsig4^+^ macrophages efficiently block the spread of gut mEVs whereas Vsig4^+^ cell population was greatly decreased in ageing mice. Gut mEV treatment resulted in cardiac inflammation and a reduction in cardiac contractility in young Vsig4^-/-^ mice. Microbial DNA depletion attenuated the pathogenic effects of gut mEVs. cGAS/STING signaling is critical for the effects of microbial DNA. Restoring Vsig4^+^ macrophage population in ageing WT mice reduced cardiac microbial DNA abundance and inflammation and improved heart contractility.

## Introduction

Ageing and the incidence of heart failure (HF) are tightly intertwined. Humans older than 65 years have an increased propensity for HF (e.g. 11/1000 persons), and the risk of HF is increased further with increased age (e.g. 43/1000 in humans > 80 years old) (1–4). This is of critical significance to the ageing population since HF is always associated with recurrent hospitalization, decreased quality of life, and a reduction in life span (5). Despite the extensive body of research, the mechanisms that determine cardiac dysfunction in ageing are incompletely understood. Consequently, translation of the findings to improve cardiac function and life span in ageing has been unsuccessful, suggesting that there is a gap in our knowledge of the molecular foundations of ageing-related cardiac impairment.

A progressive increase in the proinflammatory status referred to as “inflamm-ageing” is characteristic of the ageing process (6–12). As the hallmark of inflammageing, immune response abnormalities are evident, such as a reduction in macrophage-dependent phagocytosis (13–15). While several mechanisms such as mitochondrial disorder and senescence have been implicated in the pathogenesis of ageing-associated HF (16–19), whether the initiation and amplification of inflammation results in these cellular disorders in cardiomyocytes are unknown.

Previous studies have shown the absence of an increase in pro-inflammatory cytokines in the bloodstream and better longevity of germ-free ageing mice than their conventional counterparts, suggesting that microbiota play critical roles in promoting inflammageing phenotypes (20, 21). However, the mechanisms whereby microbiota modulates inflammageing phenotypes are not completely understood. Ageing is companied by gut barrier breach, allowing the translocation of microbiota-derived products into host circulation and distal tissues (20, 22). Previous studies have demonstrated that, in the context of gut leakage, microbiota-derived extracellular vesicles (EVs) act as important carriers spreading microbial DNA into host tissues, subsequently triggering host cellular inflammatory responses (23). Thus, these findings lead to the hypothesis that the infiltration of microbiota-derived microbial DNA containing EVs (mEVs) into the heart could cause myocardial inflammation and contractility abnormalities in ageing mice.

Here, we report that ageing-associated gut leakage and reduction of V-set and immunoglobulin domain containing 4 expressing (Vsig4^+^) macrophage population result in the translocation of gut mEVs into cardiomyocytes, leading to myocardial bacterial DNA enrichment. Bacterial DNA is the key cargo contributing to the pathogenic effects of gut EVs on inducing ageing-related cardiac abnormalities through a cGAS/STING-dependent mechanism. Finally, recovering the Vsig4^+^ macrophage population remarkably diminishes the levels of bacterial DNA in mouse ageing hearts, concomitant with decreased levels of cardiac inflammatory responses and improved myocardial contractility.

## Results

### Ageing hearts are characterized by microbial DNA accumulation

An impaired and defective intestinal barrier is observed in ageing (20, 22). Consistently, the electron microscope analysis shows that 100-108 weeks (wks) old ageing mice had an impaired gut integrity, as evidenced by increases in the length and/or diameter in adherens junctions, tight junctions, or desmosomes (**Figure S1A**). In addition, the expression of key genes associated with gut gap junction was significantly repressed in ageing mice (**Figure S1B**). After 1 hour of oral administration of FITC dextran to ageing mice, plasma FITC fluorescent intensity was markedly elevated, demonstrating increased intestinal permeability in ageing mice (**Figure S1C**). The abundance of IgA, which plays a critical role in preventing the leakage of microbiota (24), was significantly lower in ageing mouse gut, compared to young mice (**Figure S1D**). These results demonstrate that ageing is accompanied by the impairment of gut integrity.

**Figure 1:**
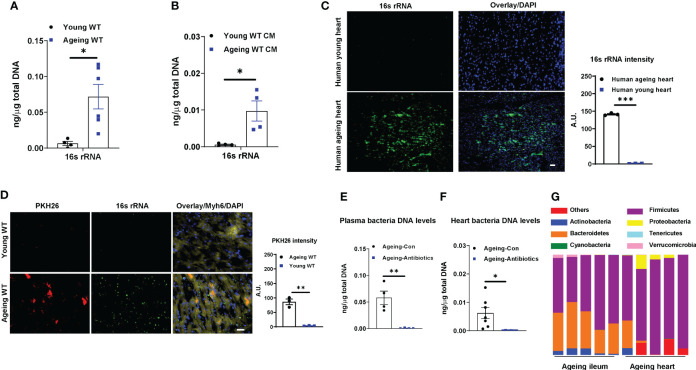
Bacterial DNA is enriched in ageing hearts. 16s rRNA abundance in plasma EVs **(A)** and cardiac myocytes (CM; **B**) isolated from young (8-12 wks old) or ageing (100-108 wks old) WT mice. **(C)** 16s rRNA levels in human hearts. Scale bar=25µm. **(D)** The appearance of PKH26 red fluorescent signals in the hearts after 16 hours of injecting PKH26-labeled gut microbial DNA containing extracellular vesicles (mEVs) into the ileum section of young or ageing WT mice. Scale bar=50µm. 16s rRNA abundance in plasma **(E)** and heart **(F)** of ageing WT mice after 2 wks of treatment with a mixture of antibiotics. Control ageing mice were treated with sterile water. **(G)** The microbial DNA species detected in ileum sections and hearts of ageing WT mice. Data are presented as mean ± SEM. *P < 0.05, **P < 0.01, ***P < 0.001, Student’s *t*-test.

Previous studies have demonstrated that microbial DNA-containing gut extracellular vesicles (mEVs) are readily translocated through an impaired gut barrier into host circulation and distal tissues (23). Consistently, the ageing-associated leaky gut resulted in the enrichment of mEVs in plasma, whereas, bacterial DNA was barely detected in the plasma EVs collected from young lean WT mice (**Figure 1A**). More importantly, as shown in **Figures 1B, C** and **S1E**, 16s rRNA was readily detected in both ageing human and mouse cardiomyocytes. To further assess whether gut mEVs are leaky from the gut lumen into host circulation and hearts in ageing mice, red fluorescent dye PKH26-labeled gut mEVs (5x10^9^ EVs/mouse) were injected into the ileum section of either young or ageing WT recipient mice. After 16 hours, PKH26 signals were readily detected in the cardiomyocytes of ageing recipient mice, thus suggesting the leakage of injected mEVs from the ageing gut (**Figure 1D**). In contrast, PKH26 fluorescence was barely detectable in young mouse hearts (**Figure 1D**).

To examine if bacterial DNA enriched in ageing heart is derived from microbiota, ageing WT mice were orally given antibiotics for 2 wks. Compared to ageing controls treated with saline, antibiotic treatment resulted in a remarkable reduction in bacterial DNA abundance in the circulation and heart (**Figures 1E, F**). We also compared the bacterial DNA species accumulated in the heart with the microbial composition of ageing WT mice. The 16s rRNA seq analysis indicates that phylum *Firmicutes* were the dominant microbial species in the ileum section of ageing gut (**Figures 1G**, **S1F–S1G**, **Tables S1**, **S2**). Consistently, the bacterial DNA enriched in ageing hearts was mainly derived from phylum *Firmicutes* (**Figure 1G**). Taken together, these data suggest that microbiota-derived mEVs are readily shuttled from the impaired intestinal barrier into the heart in the context of ageing.

### Ageing is accompanied by a loss of Vsig4^+^ macrophages

**Figure 2:**
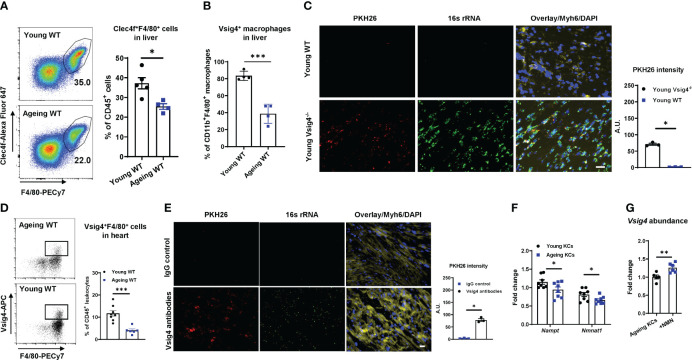
Vsig4^+^ macrophage population is remarkably decreased in ageing mice. The population of Kupffer cells (KCs, Clec4f^+^F4/80^+^; **A**) and Vsig4^+^ macrophages **(B)** in the livers of young or ageing WT mice was analyzed by flow cytometric assays. **(C)** The abundance of PKH26 signals in the hearts of young WT or Vsig4^-/-^ mice after 16 hours of tail vein injection with PKH26-labeled gut mEVs. Scale bar=50µm. **(D)** Vsig4^+^ macrophage population in hearts was analyzed by flow cytometry assays. **(E)** PKH26 intensity in hearts after 16 hours injection of gut mEVs into jugular vein of young WT mice pre-treated with either IgG or Vsig4 antibodies. Images are representative of three experiments. Scale bar=25µm. **(F)** The expression of key genes associated with NAD homeostasis in KCs was analyzed by qPCR assays. **(G)** qPCR analysis of *Vsig4* abundance in ageing KCs after *in vitro* treatment with nicotinamide mononucleotide (NMN). Data are presented as mean ± SEM. *P < 0.05, **P < 0.01, ***P < 0.001, Student’s *t*-test.

Liver Vsig4^+^ Kupffer cells (KCs) are important sentinel cells that remove bacteria and their products from the bloodstream (23, 25). We observed that ageing livers contained a significantly lower amount of Kupffer cells (Clec4f^+^F4/80^+^) than young lean mouse livers, concomitant with markedly lower Vsig4 abundance on ageing KCs (**Figures 2A, B**). Previous studies have shown that Vsig4^+^ macrophages can efficiently clean gut mEVs from the bloodstream (23). After injecting PKH26-labeled gut mEVs into the tail vein of young lean Vsig4^-/-^ mice to mimic ageing-associated high levels of mEVs in circulation, robust red fluorescent PKH26 signals were present in the heart (**Figure 2C**). We also observed that PKH26 signals were diminished after 48 hours injection and almost gone after 72 hours (**Figure S2A**). Conversely, young lean WT mice showed few PKH26 fluorescence in cardiomyocytes after 16 hours injection of PKH26 mEVs, thus demonstrating the critical role of Vsig4^+^ cells in preventing the infiltration of mEVs into the heart (**Figure 2C**).

In addition to the liver, Vsig4^+^ macrophages also reside in other tissues such as pancreatic islets and adrenal glands (26–28). Consistently, we found that ~20% of cardiac macrophages (CD11b^+^F4/80^+^) were Vsig4 positive in young mice (**Figure S2B**), whereas, ageing hearts harbored less population of Vsig4^+^ macrophages than young mice or humans (**Figures 2D**, **S2C**). In addition, ageing results in a significant reduction in the cardiac macrophage population (**Figure S2D**). To further assess the ability of cardiac Vsig4^+^ macrophages to block the infiltration of gut mEVs into cardiomyocytes, we depleted Kupffer cells by injecting diphtheria toxin (DT) to young Clec4fCre^+^DTR^+^ mice (KCKO; 10-12wks old) and then intravenously injected PKH26-labeled gut mEVs into these young KCKO mice (**Figures S2E, S2F**) (29). After 24 hours, no PKH26 signal was detected in young KCKO mice treated with PKH26 EVs (**Figure S2G**). In addition, injecting gut mEVs (5x10^8^ EVs/mouse) into the jugular vein of young WT mice did not cause bacterial DNA accumulation in the hearts (**Figure 2E**). In contrast, pre-treating Vsig4 antibodies into jugular vein blocked the function of heart Vsig4^+^ macrophages of young WT mice, subsequently resulting in the infiltration of gut mEVs into cardiomyocytes (**Figure 2E**). Thus, these data suggest that cardiac macrophages exert a profound function in cleaning gut EVs.

Previous studies have demonstrated that NAD deficiency leads to ageing-associated macrophage dysfunction (14, 30). We also found that both ageing KCs and cardiac macrophages contained less abundance of key enzymes associated with the NAD salvage pathway than young mice (**Figures 2F**, **S2H**). To assess the effect of NAD homeostasis on *Vsig4* expression, KCs isolated from ageing WT mice were treated with biosynthetic NAD precursor, NMN. After 24 hours, NMN treatment led to a significant increase in *Vsig4* abundance in ageing KCs (**Figure 2G**).

### Microbial DNA accumulation results in ageing-associated cardiac contractility defects

**Figure 3:**
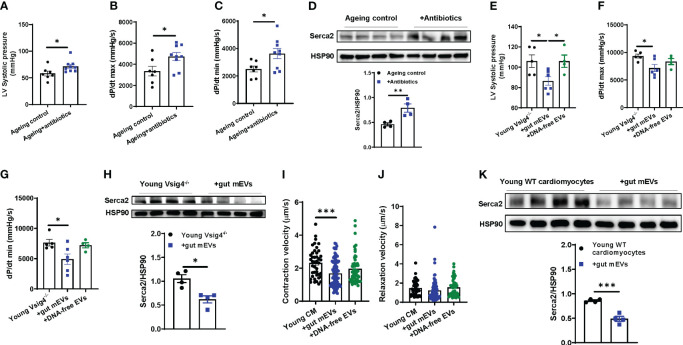
Microbial DNA accumulation impairs cardiac contractility. Left ventricular (LV) systolic pressure **(A)** maximal rate of rise of left ventricular pressure (dP/dt max; **B**) and maximum rate of ventricular pressure decrease (dP/dt min; **C**) of antibiotic-treated ageing WT mice were measured by *in vivo* cardiac contractility with the Millar catheter. **(D)** Serca2 abundance in the hearts of ageing WT mice after 2 wks of antibiotic treatment. After 4 wks of treatment with either gut mEVs or DNA-free EVs, the cardiac contractility of young Vsig4^-/-^ mice was evaluated by the levels of the left ventricular systolic pressure **(E)** maximal rate of rise of left ventricular pressure **(F)** and maximum rate of ventricular pressure decrease **(G)**. **(H)** Serca2 abundance in the hearts of young Vsig4^-/-^ mice after 4 wks of treatment of gut mEVs. After *in vitro* treatment with either gut mEVs or DNA-free EVs for 36 hours, the contractile function of young WT cardiac myocytes was evaluated by the levels of both contraction **(I)** and relaxation velocity **(J)**. **(K)** Serca2 abundance in young WT cardiomyocytes after 36 hours of treatment with gut mEVs. Data are presented as mean ± SEM. *P < 0.05, **P < 0.01, ***P < 0.001, Student’s *t*-test.

Ageing is concomitant with impaired cardiac contraction. To demonstrate the regulation of microbiota on ageing cardiac functions, ageing WT mice were treated with antibiotics (**Figure S3A**). After 2 wks of treatment, we found that depletion of microbiota led to an improvement of cardiac contraction, as demonstrated by elevated levels of left ventricular systolic pressure, the derivative of pressure over time maximum, and dP/dt (the derivative of pressure over time) minimum (min) in the hearts of antibiotic-treated ageing mice (**Figures 3A–C**). In addition, the abundance of Serca2, which is critical for cardiac calcium handling for contractility, was elevated in ageing hearts after 2 wks of antibiotic treatment (**Figure 3D**).

Given that the leakage of microbiota-derived mEVs leads to microbial DNA accumulation in cardiomyocytes in ageing mice (**Figure 1**), we next assessed the pathogenic effects of microbial DNA enrichment on cardiac functions. Young lean Vsig4^-/-^ mice were intravenously injected with gut mEVs (5x10^9^ EVs/mouse). After 4wks of mEV treatment, bacterial DNA levels in the hearts of young lean Vsig4^-/-^ mice were comparable to those of ageing WT mouse hearts (**Figure S3B**). More importantly, gut mEV treatment resulted in impaired cardiac functions, as evidenced by decreased levels of left ventricle (LV) systolic pressure, dP/dt max, and dP/dt min in the hearts of gut mEV-treated young lean Vsig4^-/-^ mice (**Figures 3E–G**). In addition, Serca2 expression was reduced in the heart after gut mEV treatment (**Figure 3H**). In contrast, depletion of microbial DNA cargo from gut mEVs led to non-significant changes in bacterial DNA levels in heart and cardiac functions of young lean Vsig4^-/-^ mice, thus demonstrating that microbial DNA is the key pathogenic cargo contributing to the effects of gut mEVs on cardiac responses (**Figures S3B**, **3E–G**). In addition, after 3wks withdrawn from gut mEV treatment, heart Serca2 levels were restored (**Figure S3C**). We also found that Vsig4 knockout didn’t affect heart phenotypes in young mice (**Figures S3D–F**).

To assess the direct effects of microbial DNA on inducing cardiomyocyte dysfunction, cardiomyocytes isolated from young WT mice were *in vitro* treated with either gut mEVs or DNA-free EVs (**Figure S3G**). Following gut mEV-induced microbial DNA accumulation, young WT cardiomyocytes displayed a marked reduction in rates of contraction (**Figures S3H**, **3I, J**). We also evaluated the state of calcium handling in cardiomyocytes after gut mEV treatment. Microbial DNA accumulation blunted the ability of young cardiomyocytes to mediate calcium homeostasis for contraction (**Figure 3K**). In contrast, depletion of microbial DNA cargos markedly reduced these effects of gut mEVs on cardiomyocyte responses (**Figures S3H**, **3I, J**). Taken together, these results demonstrate that microbial DNA is a pathogenic factor inducing ageing-associated cardiac contractility abnormalities.

### Microbial DNA enrichment induces cardiomyocyte inflammation in ageing mice

**Figure 4:**
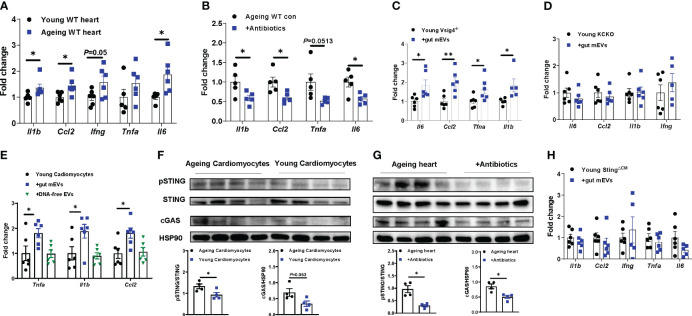
Microbial DNA enrichment triggers inflammation in cardiomyocytes. **(A)** qPCR analysis of proinflammatory gene abundance in the hearts of young vs. ageing WT mice. **(B)** Effect of antibiotic treatment on the expression of proinflammatory genes in ageing hearts. Inflammatory responses in young Vsig4^-/-^ mouse hearts **(C)** or the cardiomyocytes of young Kupffer cell depletion (KCKO) mice **(D)** after 4 wks of treatment with gut mEVs. The control young mice were treated with DNA-free EVs. **(E)** After *in vitro* treatment with gut mEVs or DNA-free EVs for 24 hours, qPCR analysis of proinflammatory gene expression in young WT cardiomyocytes. **(F)** The levels of cGAS and phosphorylated STING in cardiomyocytes isolated from young vs. ageing WT mice. **(G)** Effect of antibiotic treatment on the activation of cGAS/STING signaling in ageing WT mouse hearts. **(H)** qPCR analysis of proinflammatory gene abundance in the hearts of young cardiomyocyte-specific Sting knockout (Myh6Cre^+^Sting^f/f^, Sting^ΔCM^) mice after 4 wks of treatment with gut mEVs. Data are presented as mean ± SEM. *P < 0.05, **P < 0.01, Student’s *t*-test.

Previous studies have demonstrated that the infiltration of microbial DNA triggers mammalian cell inflammatory responses (23). Concomitant with a marked accumulation of microbial DNA in ageing hearts, ageing cardiomyocytes displayed greater levels of inflammation than young lean cardiac myocytes, as shown by a higher abundance of proinflammatory mediators, including *Il1b*, *Ccl2*, *Ifng*, and *Il6*, in ageing cells (**Figure 4A**). In contrast, microbiota-depleted ageing mice displayed attenuated inflammation in the heart, compared to ageing control mice (**Figure 4B**). We also found that heart abnormalities were restored in ageing WT mice after recovering microbiota from 2 wks antibiotic withdrawal (**Figures S3A**, **S4A, B**). To further demonstrate that microbial DNA shuttled by gut mEVs is a pathogenic factor inducing ageing-related cardiac inflammation, either young lean Vsig4^-/-^ or KCKO mice were intravenously injected with gut mEVs. The control young mice were treated with DNA-free EVs. After 4wks treatment, the hearts of young Vsig4^-/-^ mice injected with gut mEVs expressed greater levels of proinflammatory cytokines than that in control mice injected with DNA-free EVs, indicating that microbial DNA cargo within gut mEVs elevate heart inflammatory responses (**Figure 4C**). In addition, microbial DNA was barely accumulated in the cardiomyocytes of young lean KCKO mice after 4wks of intravenous treatment with gut mEVs, thus leading to comparable inflammatory responses in cardiomyocytes among young lean KCKO mice (**Figures S2F**, **4D**). After the injection of gut mEVs into jugular vein of Vsig4 antibodies-pretreated young WT mice, heart inflammation levels were elevated (**Figure S4C**). We also found that *in vitro* treatment with gut mEVs directly promoted an inflammatory state in young WT cardiac cells, whereas, depletion of microbial DNA cargo blunted these effects of gut mEVs (**Figure 4E**).

The cGAS/STING signaling is critical to sense bacterial DNA and subsequently trigger host cellular responses (31, 32). We found that ageing cardiomyocytes expressed greater levels of cGAS and phosphorylated STING (pSTING) than young cardiac myocytes, whereas, the activation of cGAS/STING signaling in the heart was blunted after depletion of microbiota and reducing heart microbial DNA accumulation in ageing mice (**Figures 4F, G**). We also demonstrated that microbial DNA shuttled by gut mEVs plays a critical role in inducing cGAS/STING signaling activation in cardiomyocytes, as shown by elevated cGAS and pSTING in gut mEVs-treated cardiomyocytes but not in DNA-free gut EV-treated cells (**Figures S4D, E**). To further assess the importance of cGAS/STING signaling on the ability of microbial DNA to induce cardiomyocyte inflammation, young lean cGAS^-/-^ (cGAS knockout) cardiac cells were treated with gut mEVs *in vitro*. After 24 hours of treatment, the cellular inflammation state was comparable among all cGAS^-/-^ cells (**Figures S4F, G**). While microbial DNA was accumulated, gut mEV treatment had minimal effects on the heart inflammation of young lean cardiomyocyte-specific Sting knockout (Myh6Cre^+^Sting^f/f^; Sting^ΔCM^) mice pre-treated with Vsig4 antibodies (**Figures 4H**, **S4H, I**). Taken together, these data suggest that microbial DNA induces ageing-related cardiac inflammation through the activation of cGAS/STING signaling.

### Microbial DNA-induced inflammation impairs cardiac contraction

**Figure 5:**
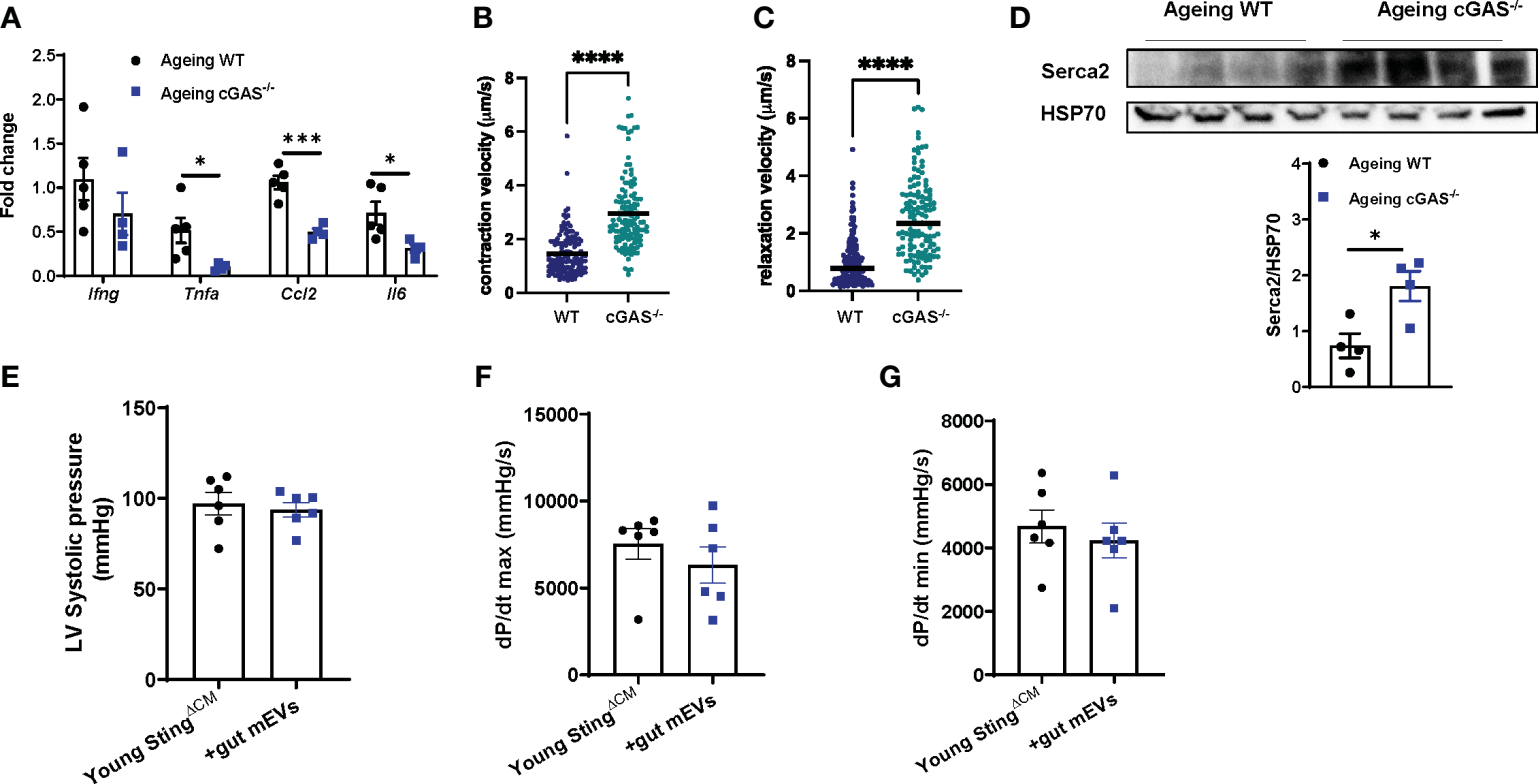
Microbial DNA-induced inflammatory responses reduce cardiac contraction capacity. The levels of proinflammatory gene expression **(A)**, contractility **(B, C)**, and Serca2 abundance **(D)** of the cardiomyocytes isolated from ageing WT vs cGAS^-/-^ mice. After 4 wks of treatment with gut mEVs, the cardiac contractility of young Sting^ΔCM^ mice was evaluated by the levels of the left ventricular systolic pressure **(E)**, maximal rate of rise of left ventricular pressure **(F)**, and maximum rate of ventricular pressure decrease **(G)**. Data are presented as mean ± SEM. *P < 0.05, ***P < 0.001, ****P < 0.0001, Student’s *t*-test.

Previous studies have demonstrated that inflammation is an important factor initiating heart dysfunctions (33, 34). While microbial DNA enrichment occurred in the cardiomyocytes of ageing cGAS^-/-^ mice, these cGAS^-/-^ ageing cardiac cells displayed lower levels of inflammation but better contractile activity than ageing WT cardiomyocytes (**Figures S5A**, **5A–C**). In addition, ageing cGAS^-/-^ cardiomyocytes contained less pSTING than ageing WT cells (**Figure S5B**). Knockout of cGAS also blunted the effects of ageing on cardiac calcium handling, as evidenced by greater Serca2 abundance in ageing cGAS^-/-^ cardiomyocytes (**Figure 5D**). We next assessed the importance of cGAS/STING-induced inflammation on the ability of microbial DNA to induce ageing-related cardiac dysfunctions. In young lean Sting^ΔCM^ mice, concomitant with their blunted inflammatory responses to microbial DNA accumulation, gut mEV treatment had minimal effects on Serca2 abundance and contractility in the heart (**Figures S4F**, **5E–G**). Thus, these data suggest that cGAS/STING-mediated inflammation plays a critical role in microbial DNA-induced ageing cardiac dysfunctions.

### Recovery of Vsig4^+^ macrophages is sufficient to attenuate ageing-associated cardiac abnormalities

**Figure 6:**
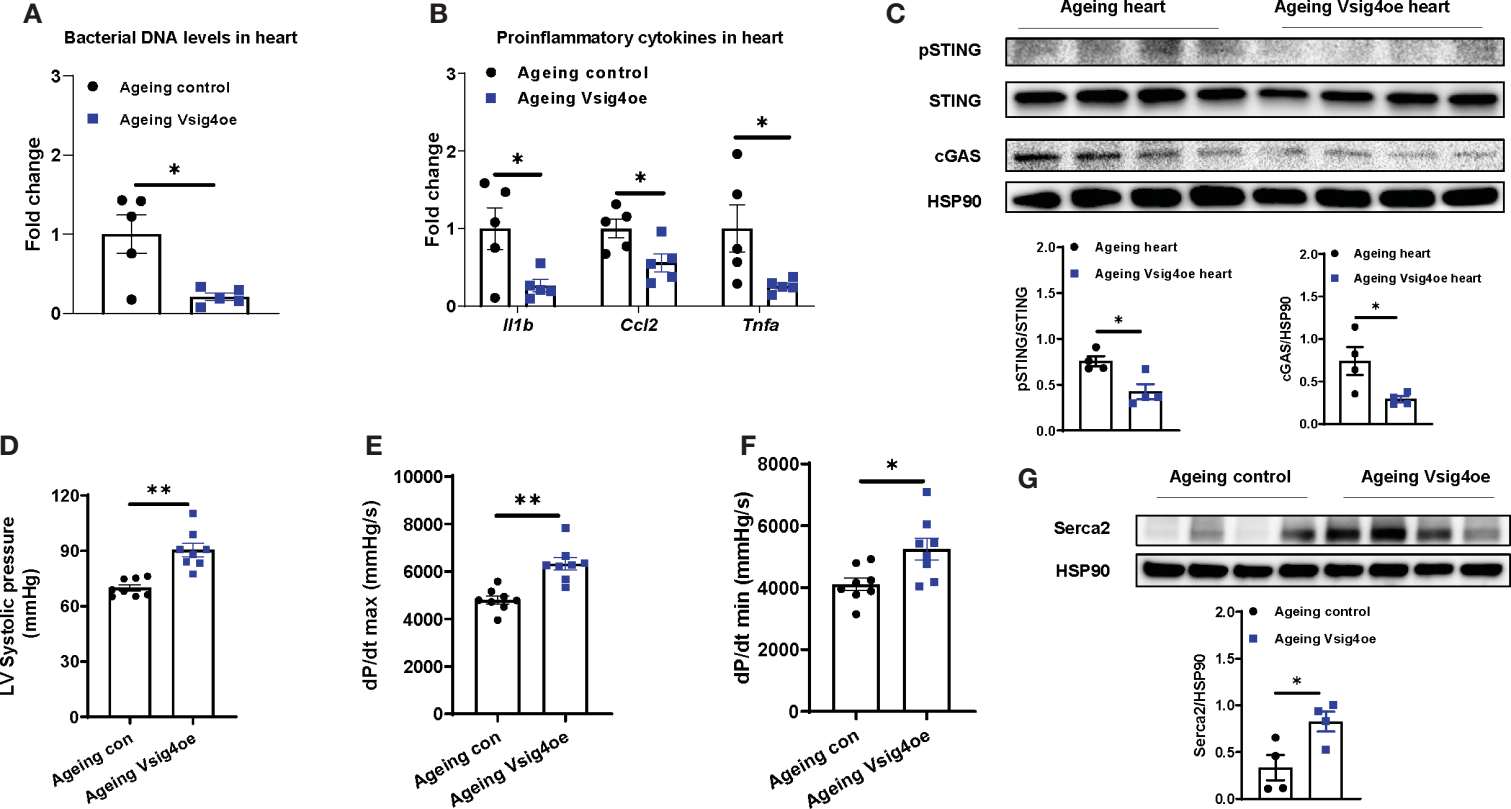
Restoring Vsig4^+^ macrophage population attenuates microbial DNA-induced cardiac abnormalities in ageing mice. The levels of bacterial DNA **(A)** and proinflammatory genes **(B)** in the hearts of ageing mice after 2 wks of injection with lentivirus carrying deactivated Cas9-VPR (VP64, p65, and Rita) and gRNA-Vsig4 transcriptional start site (TSS; Visg4oe). Control ageing mice were injected with lentivirus carrying deactivated Cas9-VPR only. After recovery of Vsig4 expression, the levels of cGAS/STING activation **(C)**, left ventricular systolic pressure **(D)**, maximal rate of rise of left ventricular pressure **(E)**, maximum rate of ventricular pressure decrease **(F)**, and Serca2 abundance **(G)** in the hearts of ageing mice were measured. Data are presented as mean ± SEM. *P < 0.05, **P < 0.01, Student’s *t*-test.

Given that Vsig4^+^ macrophages efficiently clean bacterial products from the bloodstream, we next evaluated the effects of Vsig4 overexpression on ageing-associated heart microbial DNA accumulation and cardiac functions. To overexpress Vsig4, ageing WT mice were intravenously injected with lentivirus carrying the VPR system (including three transcriptional activators VP64, p65, and Rta) linked to the C-terminal end of deactivated Cas9 (dCas9) and gRNA-Vsig4 TSS (Transcription start site; Vsig4oe). After 2 wks of injection with these lentiviruses, Vsig4 abundance was elevated in the livers and hearts of ageing mice (**Figures S6A, B**). Following the recovery of the Vsig4^+^ macrophage population, we observed that the bacterial DNA abundance was significantly reduced in the hearts of ageing mice, concomitant with lower levels of cGAS/STING activation and proinflammatory cytokines in the heart (**Figures 6A–C**). In addition, ageing mice displayed improved cardiac contraction and Serca2 abundance in the heart after restoring Vsig4^+^ macrophages (**Figures 6D–G**). Therefore, these data demonstrate that restoration of the Vsig4^+^ macrophage population is an efficient way to blunt microbial DNA-induced heart dysfunction in ageing mice.

## Discussion

In this study, we have assessed the effects of microbial DNA enrichment on the development of ageing-associated cardiac abnormalities. In specific, we find that the hearts of both ageing humans and mice harbor a robust abundance of bacterial DNA. The intestinal mEVs are readily translocated from gut lumen into the circulation and heart in ageing mice, whereas the intact gut barrier of healthy young mice prevents the leakage of intestinal mEVs. Ageing is accompanied by a significant reduction in the population of Vsig4^+^ macrophages which efficiently remove gut mEVs from the bloodstream. In the absence of Vsig4^+^ macrophages, enrichment of gut mEVs in the circulation by intravenous injection results in microbial DNA accumulation and inflammatory responses in cardiomyocytes of young Vsig4^-/-^ mice, concomitant with impaired calcium handling and cardiac contraction. In contrast, depletion of microbial DNA cargo diminishes these pathogenic effects of gut EVs on cardiac functions. The activation of cGAS/STING signaling is critical for the effects of microbial DNA, as demonstrated by non-significant changes in cardiac responses in both *in vivo* and *in vitro* experiments with cGAS or STING-KO mice after gut mEV treatment. We also demonstrate that restoring the Vsig4^+^ macrophage population remarkably decreases the levels of bacterial DNA and inflammatory responses in ageing mouse hearts, accompanied by an improvement in cardiac functions.

As one of the striking phenotypes of this study, both human and mouse hearts contained a great abundance of bacterial DNA, which was barely detected in healthy young hearts. In addition, ageing bloodstream was enriched with bacterial DNA. Previous studies have suggested that microbiota could be the origins of bacterial DNA accumulated in host tissues in the scenario of the impaired gut barrier, for example, obesity-induced gut leakage (23, 35, 36). Indeed, the 16s rRNA sequencing analysis indicates that the bacterial DNA profile in ageing hearts shares some similarities, for example, phylum *Firmicutes*, with the microbiome composition in ageing ileum section. The microbiota in other intestinal sections may also contribute to the microbial DNA accumulation in ageing heart. It would be interesting to evaluate whether circulating bacterial DNA species could be related to the development of ageing-associated abnormalities. In addition, gut barrier breach occurs in both ageing humans and mice (12), thus likely allowing the leakage of microbiota-derived products into host circulation and distant organs. The impairment of ageing-related gut barrier functions could be due to reduced mucus thickness and functions and abnormal intestinal structure (22, 37, 38). In concordance with these earlier findings, we also observed decreased IgA abundance and impaired gut barrier structure. Previous studies have demonstrated that, in the context of obesity-induced gut leakage, microbiota-derived mEVs are readily translocated into the host bloodstream and various tissues (23, 26, 27). Consistently, we found that the ageing mouse gut barrier did not efficiently block the leakage of gut mEVs, as evidenced by strong red fluorescent signals in the hearts after an injection of PKH26-labeled gut EVs into the ileum section of ageing WT mice. With respect to the manners in which gut mEVs interact with target cells, several mechanisms, including integrin-dependent tropism and endocytosis, have been implicated (39, 40). However, how microbiota-derived EVs enter host cells is still unknown.

In addition to the protection of the gut barrier, the complement immune system plays critical roles in cleaning up bacterial products from the bloodstream in both humans and mice (25, 41). Vsig4^+^ macrophages are important components of the host complement immunity (25). Previous studies have demonstrated that Vsig4^+^ macrophages can prevent the spread of gut mEVs into host tissues (23). While Vsig4^+^ macrophages mainly reside in the liver, we also found that a portion of cardiac macrophages was Vsig4 positive in the young heart. In the young KCKO mouse models, an intravenous injection of gut mEVs did not efficiently infiltrate into hearts, suggesting the protective roles of cardiac Vsig4^+^ macrophages. However, we found that the population of Vsig4^+^ macrophages in both liver and heart was diminished in ageing humans and mice. It has been reported that the impairment in NAD homeostasis and metabolism contributes to the functional abnormalities of ageing macrophages (14, 30, 42). In our studies, we also observed a significant reduction in the expression of key enzymes involving the NAD salvage pathway in ageing KCs and cardiac macrophages. In addition, supplementation of NMN restored Vsig4 expression in ageing macrophages, suggesting the importance of NAD homeostasis for macrophages to maintain Vsig4 abundance. The beneficial effects of NAD homeostasis or Vsig4^+^ macrophage restoration should be further validated *in vivo* in both ageing humans and mice. Interestingly, in 2 years old chromogranin A deficient mice, liver Vsig4 abundance were greater than WT ageing mice, accompanied by less bacterial DNA abundance and remarkable reduction in bacterial DNA-induced inflammation (43). A previous study by Hall et al. observed an increase in Vsig4+ cell population in the fat pad of ageing mice (44). The discrepancy in Vsig4 abundance in ageing liver, fat, and heart may be attributed to the distinct tissue microenvironment.

Ageing heart is characterized by elevated inflammatory responses, which result in impaired cardiac contractility (19, 45). In this study, we have shown that the treatment of gut mEVs in young Vsig4^-/-^ mice led to the accumulation of microbial DNA in cardiomyocytes, concomitant with an elevated cellular inflammatory response and reduced contractility. In contrast, depletion of microbial DNA cargo abolished these effects of gut EVs, thus demonstrating the pathogenic roles of microbial DNA in inducing ageing-associated cardiomyocyte inflammation and abnormal contraction. In line with the importance of cGAS/STING signaling activation on the ability of microbial DNA in host cells (31, 32), knockout of either cGAS or STING minimized the pathogenic effects of microbial DNA accumulation on cardiac myocyte responses. Previous studies have shown that cellular senescence is a causal factor driving ageing-related cardiac inflammation (46, 47). However, the connection between the microbial DNA-induced cGAS/STING activities and the incidence of cellular senescence in cardiac myocytes is still unclear. We also observed that microbial DNA accumulated in other cell types in the ageing heart. The effects of microbial DNA on these non-cardiomyocyte cell responses would be explored in future studies.

In summary, we find that microbial DNA is transported by EVs from the gut lumen into distant cardiomyocytes in the context of ageing, resulting in elevated cardiac inflammation and contractility defects. Vsig4^+^ macrophages in both the liver and heart are critical to prevent microbial DNA accumulation in cardiomyocytes, whereas ageing macrophages display a significant reduction in Vsig4 expression. Recovery of Vsig4 expression effectively attenuates the pathogenic impacts of microbial DNA on cardiac functions in ageing mice. Based on these studies, we suggest a new mechanism whereby microbial DNA accumulation induces cardiac inflammation and decreased contractility in ageing.

## Methods

### Animal care and use

cGAS^-/-^ (Stock No. 026554) Sting-flox (Stock No. 031670), iDTR (Stock No. 007900; Cre-inducible expression of diphtheria toxin receptor), Myh6-Cre (Stock No. 011038), Clec4f-Cre (Stock No. 033296) mice were received from the Jackson Laboratory. Vsig4^-/-^ mice (C57BL/6J-Vsig4^em1Smoc^) were received from Shanghai model organisms. Vsig4 wild-type (WT) mice were produced by crossing Vsig4 heterozygous mice together. Ageing mice used in these studies were 100-108 wks old, and young mice were 8-12 wks old. To block Vsig4 function, young Myh6Cre^+^Sting^f/f^ male mice were injected with purified Vsig4 antibodies before gut mEV treatment. through tail vein injection. All mice were maintained on a 12/12 hr light-dark cycle. All animal procedures were performed in accordance with the University of California, San Diego Research Guidelines for the Care and Use of Laboratory Animals, and all animals were randomly assigned to cohorts when used.

### EV purification and characterization

The intestinal EVs were prepared from small intestine lumen contents of ageing WT mice with sterile tools. Debris and dead cells in the lumen contents were removed by centrifugation at 1,000 x g for 10 min and then passed through a 0.2 µm filter. Then, the supernatant was added with a mixture of antibiotics (0.5 mg/mL vancomycin HCl, 1 mg/mL ampicillin sodium salt, 1 mg/mL metronidazole, 1 mg/mL neomycin sulfate, and 1mg/mL gentamycin sulfate; Sigma) and then ultracentrifuged at 100,000 x g for 4 hours at 4°C with a Type 70 Ti fixed-angle rotor (Beckman Coulter). The EV-containing pellet was resuspended in 1 mL of sterile PBS and passed through a 0.2 µm filter to remove large particles in a sterile hood. The particle size and concentration of intestinal EVs were detected by NanoSight analysis (Malvern Instruments).

### Red fluorescent dye PKH26-labeled gut mEVs

PKH26 fluorescent dye using the PKH26 fluorescent cell linker kit (Cat. No. PKH26GL-1KT, Sigma). After PKH26 staining, the EVs were washed with sterile PBS and collected by ultracentrifugation (100,000 x g for 2 hours) at 4°C. Finally, PKH26 labeled EVs were resuspended in sterile PBS and passed through a 0.2 µm filter.

### *In vivo* EV trafficking assays

PKH26-labeled EVs (5x10^9^ EVs per mouse) were delivered to either young or ageing WT recipient mice through injection into the ileum section. After anesthetized, the ileum section was exposed through making a small incision at the abdominal area, and then 150 µL of saline containing EVs were injected into ileum. Following closure of incisions, sites will be treated with 10% povidone-iodine solution. After 16 hours of injection, hearts were collected to detect PKH26 red fluorescent signals.

### Jugular vein cannulation and antibody injection

Mice will be anesthetized with ketamine (25 mg/kg), acepromazine (1 mg/kg) and xylazine (2 mg/kg) via intramuscular injection. Incision sites will be shaved and cleaned with isopropyl alcohol and a 10% povidone-iodine solution. The right jugular vein will be cleared of surrounding tissue and a sterilized dual microreathane catheter (Type MRE-025) filled with heparinized saline (100 U/mL) will be inserted ~1 cm into vessel and secured with double silk ligatures. Catheter ends will then be tunneled subcutaneously to the mid-scapular region, externalized, placed in a protective silastic tubing (0.18 cm), and sutured to the skin. Following closure of incisions, sites will be treated with 10% povidone-iodine solution and animals will be given 1cc 0.9% sterile saline subcutaneously for rehydration. Lidocaine (2.5%) will be administered twice a day for 5 days post-surgery. To block the function of Vsig4+ macrophage in hearts, Vsig4 antibodies (0.5 mg/ml) were injected into heart through the jugular vein catheter. Control mice were injected with IgG antibodies. After 24 hours, PKH26-labeled gut mEVs (5x10^8^ EVs/mouse) were injected into the jugular vein catheter of these antibodies-treated mice.

### *In vivo* FITC dextran assay

Mice were fasted for 6 hours and then orally gavaged with FITC dextran (600mg/Kg body weight; Sigma) from a 125mg/mL solution (48, 49). Plasma samples were collected after 1 hour FITC dextran treatment and used to measure the intensity of FITC by fluorescence spectroscopy (excitation at 485nm and emission at 535nm) relative to a linear standard curve made using diluted FITC dextran solution in plasma from untreated mice.

### Depletion of microbial DNA cargo from gut EVs

The isolated gut EVs from ageing WT mice were resuspended in sterile PBS. Then, as previously described (23, 50–52), these EVs were loaded into a Gene Pulser/micropulser Cuvettes (Bio-Rad) for electroporation (GenePulser Xcell electroporator, Bio-Rad) and treated with DNase I (300U) for 30 mins, 37°C.

### Isolation of adult ventricular cardiomyocytes

Calcium-tolerant adult cardiomyocytes were isolated from ventricular tissue of mice by standard enzymatic digestion (53, 54). Briefly, isolated hearts were perfused using a Ca^2+^-free Tyrode solution containing (in mM) 126 NaCl, 4.4 KCl, 1.2 MgCl_2_, 0.12 NaH_2_PO_4_, 4.0 NaHCO_3_, 10 HEPES, 30 2,3-butanedione monoxime, 5.5 glucose, 1.8 pyruvate, and 5.0 taurine (pH 7.3) for 5 min, followed by 0.9–1.0 mg/mL collagenase (type II, Worthington) for 20 min. Hearts were transferred to tubes containing fresh collagenase for an additional 10 min in a 37°C water bath. The heart tissue was mechanically dispersed and rinsed with gradually increasing extracellular calcium to 1 mM. The cells were plated on 24 × 50-mm, No. 1 glass coverslips coated with laminin.

### Invasive hemodynamic measurements

After anesthetized and endotracheal intubation, mice were connected to a volume-cycled rodent ventilator. The anterior neck and abdomen were shaved. A subcostal incision was performed in the abdomen. The diaphragm was incised by a transverse substernal approach leaving the pericardium intact. The left ventricle was entered through an apical stab with a 25 1/2 G needle, followed immediately by a 1F Millar conductance-micromanometer (Millar Instruments). The catheter was positioned in the left ventricle and monitored to verify correct placement. Pressure recordings were obtained and analyzed to obtain left ventricular pressure (LVP), rate of contraction (dP/dt max) and rate of relaxation (dP/dt min).

### Contractility measurements *in vitro*

Cardiac myocyte (CM) contractility is measured with a state-of-the-art integrated myocyte contractility workstation (IonOptix LLC). This method combines brightfield imaging with a high-speed camera recording system, along with sarcomere and cell-length detection algorithms, and application-specific analysis software to output reliable quantification of cardiomyocyte contractile function at the sarcomere level. Analysis outputs include determination of ±ΔL/Δt (dL/dt), representing peak shortening/relengthening velocities, and percent shortening for both sarcomere and cell length. CM contractility was measured after 36 hours of treatment with gut EVs.

### *In vitro* assays with Kupffer cells

KCs (Clec4f^+^F4/80^+^) isolated from ageing WT mice were seeded in a 24-well plate (0.5x10^5^/well) and cultured with nicotinamide mononucleotide (NMN; 1mM). After 24 hours, cells were used for qPCR assays.

### 16s rRNA sequencing and bioinformatics analysis

Bacterial DNA was isolated from ileum lumen content and heart samples using the ZymoBIOMICS DNA extraction kits. Bacterial 16s ribosomal RNA gene targeted sequencing was performed using the Quick-16S™ NGS Library Pre Kit (Zymo Research). The bacterial 16s primers amplified the V3-V4 region of the 16s rRNA gene. The final PCR products were quantified with qPCR fluorescence readings and pooled together based on equal molarity. The final pooled library was cleaned with the Select-a-Size DNA Clean & Concentrator™ (Zymo Research), then quantified with TapeStation^®^ (Agilent Technologies) and Qubit^®^ (Thermo Fisher). The final library was sequenced on an Illumina MiSeq with a v3 reagent kit (600 cycles). The sequencing was performed with 10% PhiX spike-in. For bioinformatics analysis, unique amplicon sequences variants were inferred from raw reads using the DADA2 pipeline (55). Potential sequencing errors and chimeric sequences were also removed with the DADA2 pipeline. Chimeric sequences were also removed with the DADA2 pipeline. Taxonomy assignment was performed using Uclust from Qiime v.1.9.1 with the Zymo Research Database, a 16s database that is internally designed and curated. Composition visualization, alpha-diversity, and beta-diversity analysis were performed with Qiime v.1.9.1 (56). If applicable, a taxonomy that has significant abundance among different groups was identified by LEfSe using default settings (57). Sequencing raw data supporting these studies can be found in the Sequence Read Archive database under accession number PRJNA859808.

### Kupffer cell-depleted (KCKO) mice

To deplete Kupffer cells, diphtheria toxin (DT) was intraperitoneally (i.p.) injected into Clec4fCre^+^DTR^+^ lean mice (crossbreeding Clec4fCre mice and iDTR mice) daily for three days (200 ng/mouse). Thereafter, these mice were injected with DT (200 ng/mouse) every two days to prevent KC recovery (26).

### CRISPR-Cas9 system for transcriptional activation

Plasmids containing deactivated Cas9-VPR (VP64, p65, and Rta) system (Cat. No. CAS11915) or guide RNA for Vsig4 TSS (Cat. No. GSGM11893-247477006) were obtained from Horizon Discovery (58–60). The lentivirus packing these plasmids were prepared by the UCSD Vector core. 100wks old ageing WT mice were intravenously injected with these lentiviruses (1x10^8^ particles/mouse). Control mice were treated with lentivirus containing dCas9-VPR only. After 2 wks, Vsig4 expression in livers and hearts was evaluated by Western blot analysis.

### *In vivo* antibiotic treatment

Ageing WT mice (100-108wks old) were subject to oral gavage (twice per week; 200 µL per mouse) with a mixture of broad-spectrum antibiotics (0.5 mg/mL vancomycin HCl, 1 mg/mL ampicillin sodium salt, 1 mg/mL metronidazole, 1 mg/mL neomycin sulfate, and 1mg/mL gentamycin sulfate; Sigma) (48). Control mice were treated with water alone. The ageing WT mice in the antibiotic withdrawal group were fed ad libitum without antibiotic treatment for another 2 wks.

### Quantification of bacterial DNA using real-time PCR

Bacterial DNA was assessed by qPCR using a Femto Bacterial DNA Quantification Kit by following the manufacturer’s instructions. Briefly, Bacterial DNAs were extracted from cells, tissues, or plasma samples using the ZymoBIOMICS DNA extraction kits according to the manufacturer’s instructions. The concentration of bacterial DNA in each sample was calculated from the standard curve.

### Immunofluorescence staining

Mouse hearts were snap frozen in optimum cutting temperature (O.C.T., Fisher Healthcare) with dry ice. Tissue cryo-sections were prepared and fixed with pre-cold acetone for 20 min. Slides were blocked with 5% normal donkey serum for 60 min at RT. Then, the samples were incubated with antibodies diluted 1:100 in PBS at 4°C overnight. After washing, nuclei were stained with DAPI (4’,6-Diamidino-2-28 phenylindole dihydrochloride) for 10min at room temperature. Mounting media and coverslips were then added to slides for imaging. Images were acquired on a Keyence Fluorescent Microscope and were processed with ImageJ (NIH, Bethesda, MD).

### RNAscope *in situ* hybridization combined with immunofluorescence

We performed RNAscope ISH to detect 16s rRNA. Mouse hearts were frozen in O.C.T with dry ice. Human heart sample information is presented in **Table S3**. Tissue sections were fixed with 4% PFA for 15 min at 4°C and then dehydrated with 50%, 70%, and 100% ethyl alcohol gradients for 5 min each at room temperature. Next, tissue sections were treated with hydrogen peroxide and protease IV at room temperature for 10 min each. 16s RNA probes (Cat. No. 464461, Advanced Cell Diagnostics) were then added for 2 hours at 40°C. Signal amplification and detection reagents were applied sequentially and incubated in AMP 1, AMP 2, AMP 3, HRP-C1 (RNAscope® Multiplex fluorescent reagent kit v2, Cat. No. 323100, Advanced Cell Diagnostics), Opal 520 (Cat. No. PNFP1487001KT, Akoya Biosciences), or Opal 690 (Cat. No. FP1497001KT, Akoya Biosciences). Then, slides were immediately processed for immunofluorescence, and images were taken with a Leica SP8 Confocal microscope.

### Flow cytometry analysis

The liver and heart were perfused to get single cells which were then stained with fluorescence-tagged antibodies. KCs were CD45^+^Clec4f^+^F4/80^+^ cells, and cardiac macrophages were CD45^+^CD11b^+^F4/80^+^ cells. These cells were analyzed by an MA900 flow cytometer (SONY). Data were analyzed using Flowjo software. Clec4f (Cat. No. 156804), F4/80 (Cat. No. 123114), CD11b (Cat. No. 101235), and CD45 (Cat. No. 103116) antibodies were received from Biolegend, and Vsig4 antibody (Cat. No. 17-5752-82) was from ThermoFisher Scientific. The concentration of antibodies (0.25µg per 10^6^ cells) for sample staining were used.

### Quantitative reverse transcriptase-polymerase chain reaction analysis

Total RNA was extracted using the RNA extraction protocol according to the manufacturer’s instructions. cDNA was synthesized using MultiScribe Reverse Transcriptase and random primers (High-capacity cDNA reverse transcription kit, Cat. No. 4368813, ThermoFisher Scientific). qPCR was carried out in 10 μl reactions using iTaq SYBR Green supermix (Cat. No. 172-5125, Bio-Rad) on a StepOnePlus Real-Time PCR Systems (ThermoFisher Scientific). The data presented correspond to the mean of 2-ΔΔCt from at least three independent experiments after being normalized to β-actin.

### Western blot analysis

Cells or tissues were homogenized in 1x RIPA buffer supplemented with protease and phosphatase inhibitors. Equal amounts of cell lysate proteins (30 μg protein per lane for detection) from each biological replicate were subjected to western blotting. Using the ChemiDoc XRS imaging system (BioRad), the protein bands on blots were detected with the SuperSignal West Pico Chemiluminescent Substrate (Cat. No. 34077, ThermoFisher Scientific). Protein bands were analyzed using Image Lab software (BioRad). We normalized phosphorylated protein to total protein bands or normalized protein expression to housekeeping protein bands. Western blot data in figures and supplemental figures are all representatives of more than three independent experiments. pSTING (Cat. No. 72971), STING (Cat. No. 50494), cGAS (Cat. No. 316595), and Serca2 (Cat. No. 4388S) antibodies were bought from Cell Signaling Technology. Vsig4 antibody (Cat. No. 17-5752-82) was obtained from ThermoFisher Scientific. All primary antibodies were diluted at 1:1000.

### Lentivirus production

Production of lentivirus vectors: HIV1-based lentivirus vectors were produced by transient co-transfection of 293T cells maintained in Dulbecco’s modified Eagle’s medium (DMEM) with 10% FCS. 293T cells in ten 150 mm dishes were co-transfected by the polyethyleneimine method with gRNA vector plasmid, pLP1 (gal-pol) and pLP2 (Rev) (Invitrogen), and pCMV-G (61). Conditioned media on day 1, 2, and 3 post-transfection were collected, filtered through a 0.45 µm filter, and concentrated by centrifugation at 7000 rpm for 16 hours at 4°C with Sorvall GS-3 rotor. The resulting pellets were resuspended with buffer containing 10 mM Tris HCl, pH 7.8, 1 mM MgCl_2,_ and 3% sucrose. Titering of HIV1 vectors by real-time Q-PCR: HIV1-CMV-GFP vector (1x10^9^ iu/mL) was used as the standard. HEK293 cells in a 6-well plate were infected with different amounts of viruses in the presence of polybrene (4 µg/mL). Infected cells were passaged once every 4 days and cell DNAs were prepared on day 14 post-infection by the DNeasy Blood & Tissue kit (Qiagen Science, MD). Real-time Q-PCR was performed using a primer set selected from the WPRE sequence.

### Statistical analysis

To assess whether the means of two groups are statistically different from each other, an unpaired two-tailed Student’s *t*-test was used for statistical analyses using Prism8 software (GraphPad software v8.0; Prism, La Jolla, CA). P values of 0.05 or less would be considered to be a significant difference. Degrees of significance was indicated in each of the figure legends.

## Data availability statement

The datasets presented in this study can be found in online repositories. The names of the repository/repositories and accession number(s) can be found below: PRJNA859808 (SRA).

## Ethics statement

The animal study was reviewed and approved by University of California, San Diego Research Guidelines for the Care and Use of Laboratory Animals.

## Author contributions

WY and JS designed the studies and HG, KW, and JAS performed most of the experiments. ZJ, AF, TT, YT, BT, HJ, JK, assisted with mouse genotyping and tissue collection. KR performed jugular vein cannulation. ZL assisted with ISH staining. SM helped with EM and imaging. WD, JS, and WY supervised the project. HG, KW, JAS, WD, JS, and WY analyzed and interpreted the data, and HG, JAS, JS, and WY wrote the manuscript. All authors contributed to the article and approved the submitted version.
