# Supplementary material for: Gut lumen-leaked microbial DNA causes myocardial inflammation and impairs cardiac contractility in ageing mouse heart
Source: Front Immunol. 2023 Jul 13;14:1216344. doi: 10.3389/fimmu.2023.1216344 (PMC10373503; doi:10.3389/fimmu.2023.1216344)
Supplement: Supplementary file 1 [file DataSheet_1.pdf]

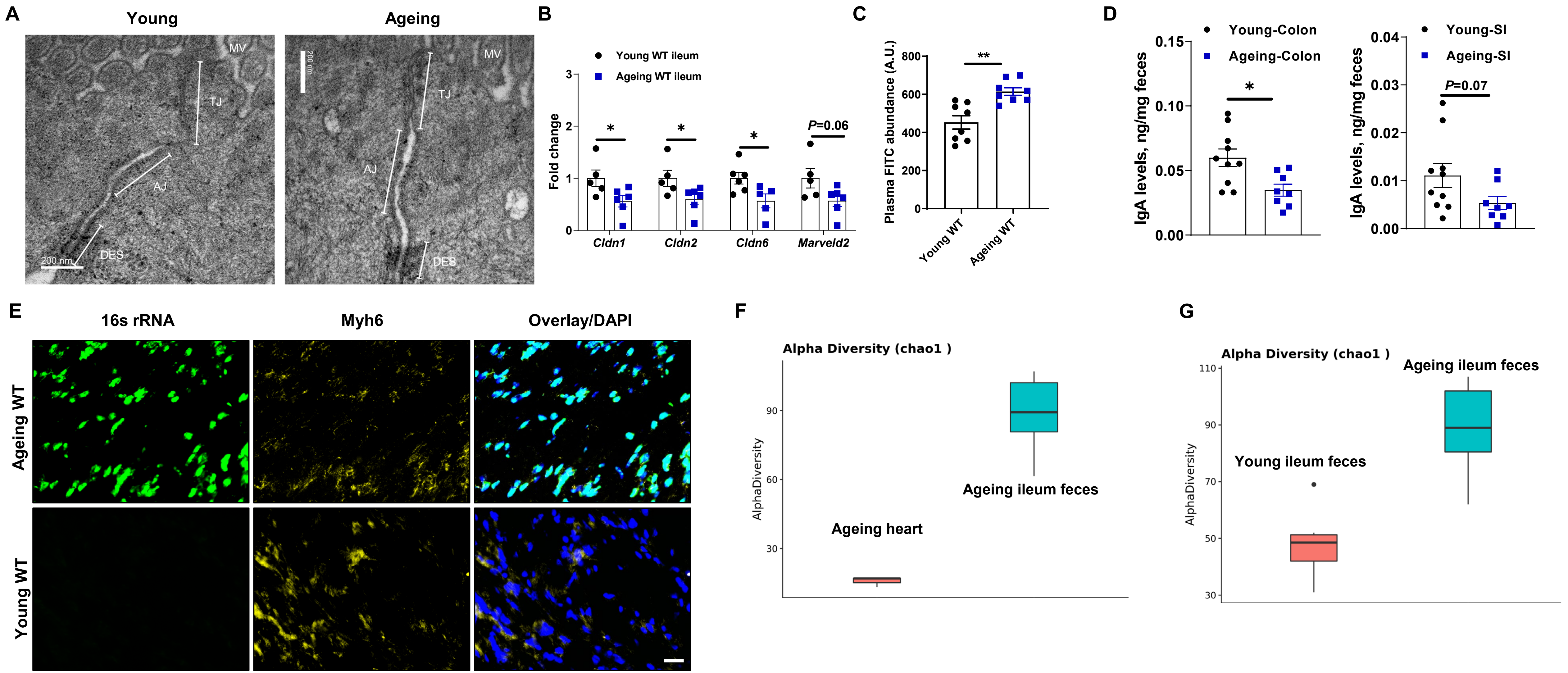

Figure S1. Ageing mice are characterized by the gut barrier breach. **A**, The barrier structure of ileum sections of young vs. ageing WT mice measured by electron microscopy analysis. TJ, tight junction; AJ, adherens junction; DES, desmosome; MV, microvillus. **B**, qPCR analysis of key genes associated with gut barrier integrity in ileum sections of young vs. ageing WT mice. **C**, The levels of FITC intensity in plasma of either young or ageing WT mice after 1 hour orally gavaged with FITC dextran. **D**, IgA abundance in the lumen contents of colon and small intestine (SI) of young vs. ageing WT mice. **E**, 16s rRNA abundance in the hearts of young and ageing WT mice. Scale bar=50μm. Alpha diversity analysis between the hearts and ileum feces of ageing WT mice (**F**) or Young vs. Ageing ileum feces (**G**). Data are presented as mean ± SEM. *P* values are determined by Student's *t*-test.

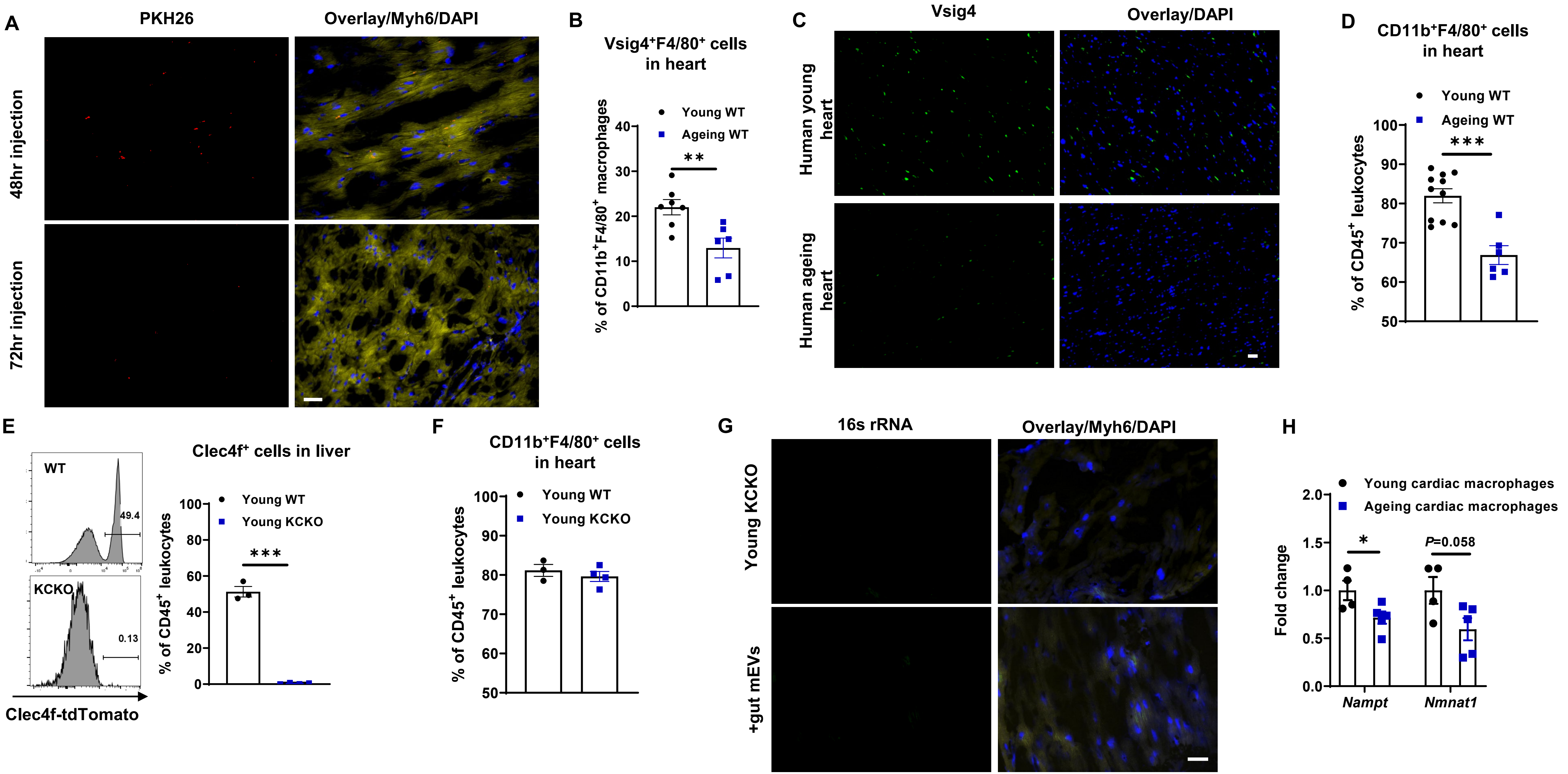

Figure S2. Vsig4 expression is repressed in the macrophages of ageing mice. **A,** PKH26 signals in the heart of young Vsig4$^{-/-}$ mice after tail-vein injection of gut mEVs. **B**, Flow cytometry analysis of Vsig4$^+$ macrophage population in the hearts of young vs ageing WT mice. Scale bar=50µm. **C**, Vsig4 abundance in human hearts. Scale bar=25µm. **D**, Flow cytometry analysis of cardiac macrophage (CD11b$^+$F4/80$^+$) population in young vs. ageing WT mice. Clec4f$^+$ cell population in the liver (**E**) and heart macrophages (**F**) of Clec4fCre$^+$DTR$^+$ mice vs. Clec4fCre$^+$ DTR$^-$ mice after 24hr i.p. injection of DT. **G**, 16s rRNA abundance in the hearts of young Kupffer cell-depleted mice (KCKO) after 16 hours of tail vein injection with gut mEVs. Images are representative of three experiments. Scale bar=50µm. **H**, qPCR analysis of key genes associated with NAD homeostasis in cardiac macrophages. Data are presented as mean ± SEM. *P* values are determined by Student's *t*-test.

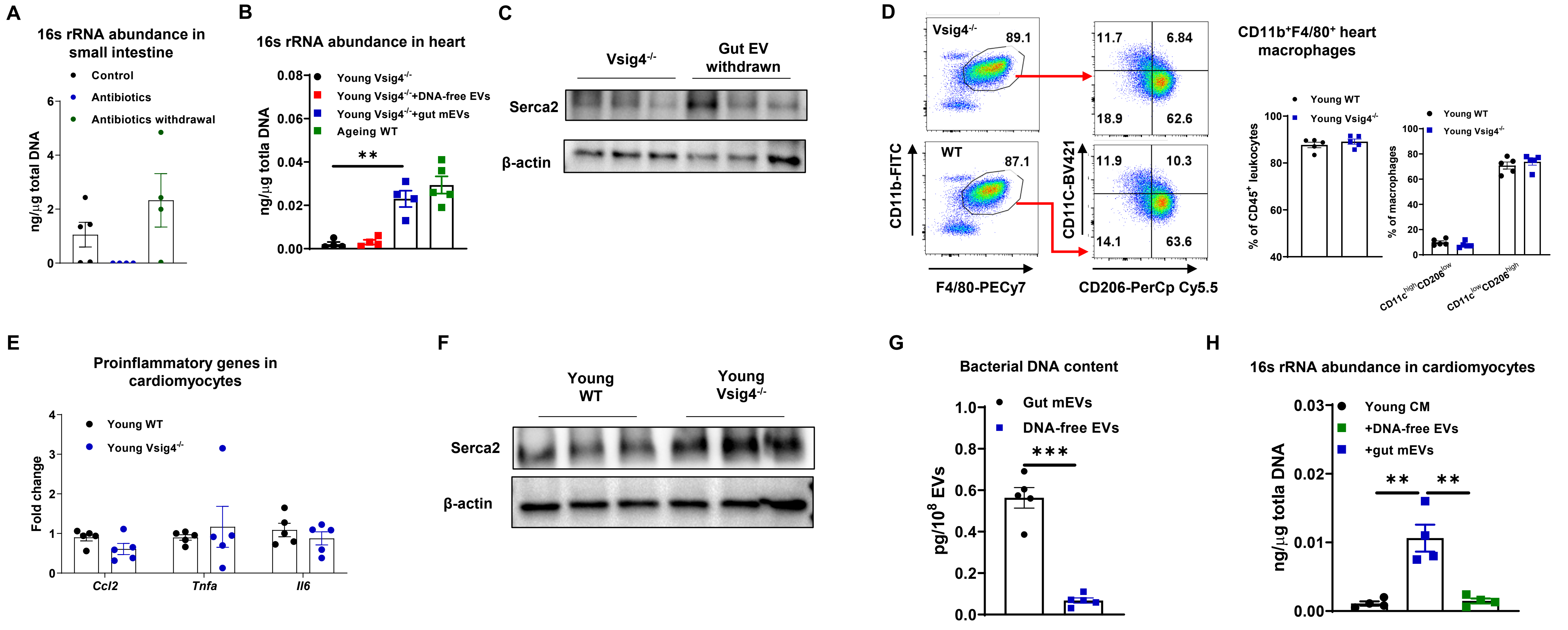

Figure S3. The importance of bacterial DNA cargo on the effects of gut mEVs. **A**, Bacterial DNA abundance in small intestine of ageing WT mice after 2wks antibiotics treatment. A group of antibiotics-treated ageing mice was withdrawn from antibiotics treatment for another 2 weeks (antibiotics withdrawal). **B**, qPCR analysis of 16s rRNA abundance in the hearts of young Vsig4$^{-/-}$ mice after injection with either gut mEVs or DNA-free EVs. **C**, Heart Serca2 abundance of young Vsig4$^{-/-}$ mice withdrawn from gut mEV treatment. Heart macrophage phenotypes (**D**), cardiomyocyte inflammatory gene expression (**E**), and Serca2 abundance (**F**) in the hearts of 8wks old WT vs. Vsig4$^{-/-}$ mice. **G**, qPCR analysis of bacterial DNA content in gut mEVs after DNase digestion. **H**, qPCR analysis of 16s rRNA levels in young WT cardiomyocytes after treatment with gut mEVs or DNA-free EVs. Data are presented as mean ± SEM. *P* values are determined by Student's *t*-test.

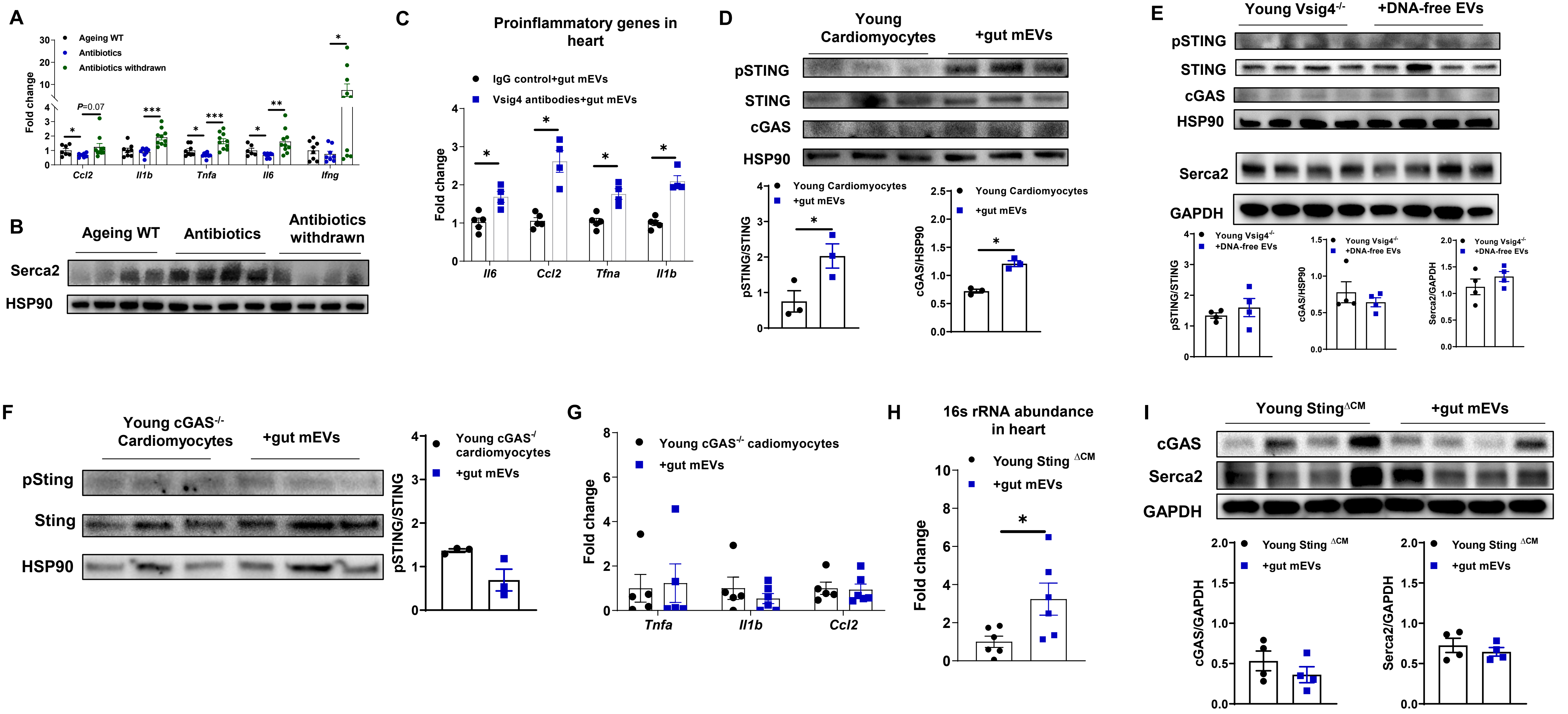

Figure S4. cGAS/STING signaling is critical for the effects of gut mEVs. Heat inflammation levels (**A**) and Serca2 abundance (**B**) in ageing WT mice after 2wks antibiotics withdrawal. **C**, Proinflammatory gene expression in the hearts of Vsig4 antibodies-treated young WT mice after jugular vein injection of gut mEVs. **D**, The levels of cGAS and phosphorylated STING in young WT cardiomyocytes after treatment with gut mEVs. **E**, Effects of DNA-free EVs on cGAS/STING and Serca2 abundance in the hearts of young Vsig4$^{-/-}$ mice. The levels of phosphorylated STING (**F**) and proinflammatory genes (**G**) in young cGAS$^{-/-}$ (cGAS knockout) cardiomyocytes after *in vitro* treatment with gut mEVs. The expression of 16s rRNA (**H**) and cGAS and Serca2 (**I**) in the hearts of young cardiomyocyte-specific Sting knockout (Sting$^{\Delta CM}$; Myh6Cre^{+}Sting$^{f/f}$) mice after 4 weeks treatment of gut mEVs. Data are presented as mean ± SEM. *P* values are determined by Student's *t*-test.

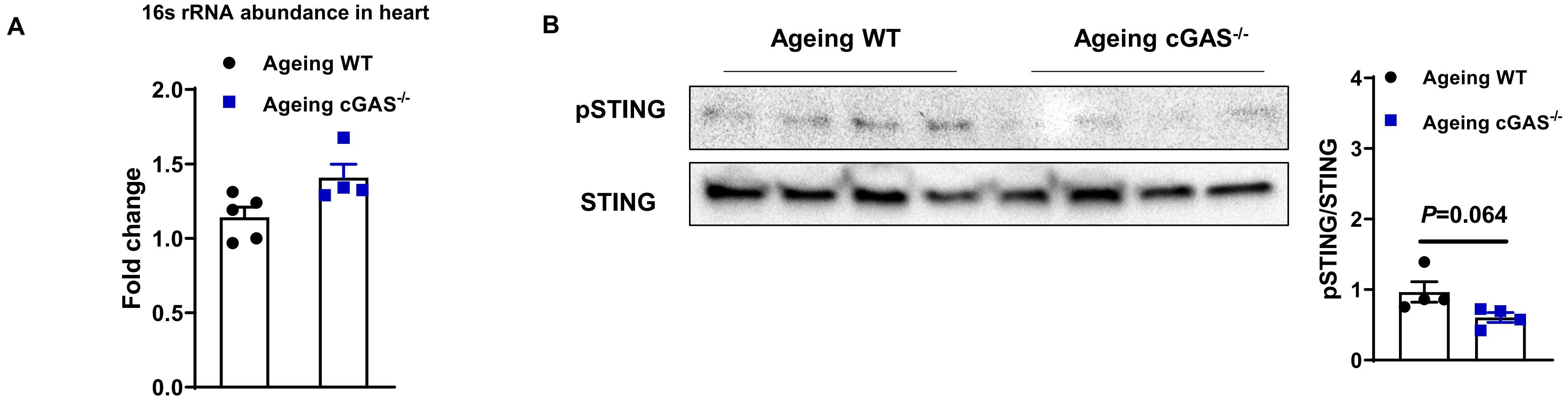

Figure S5. The inflammatory phenotypes in the hearts of ageing cGAS$^{-/-}$ mice. The abundance of 16s rRNA in hearts (**A**) and phosphorylated STING in cardiomyocytes (**B**) of ageing WT vs. cGAS$^{-/-}$ mice. Data are presented as mean ± SEM. *P* values are determined by Student's *t*-test.

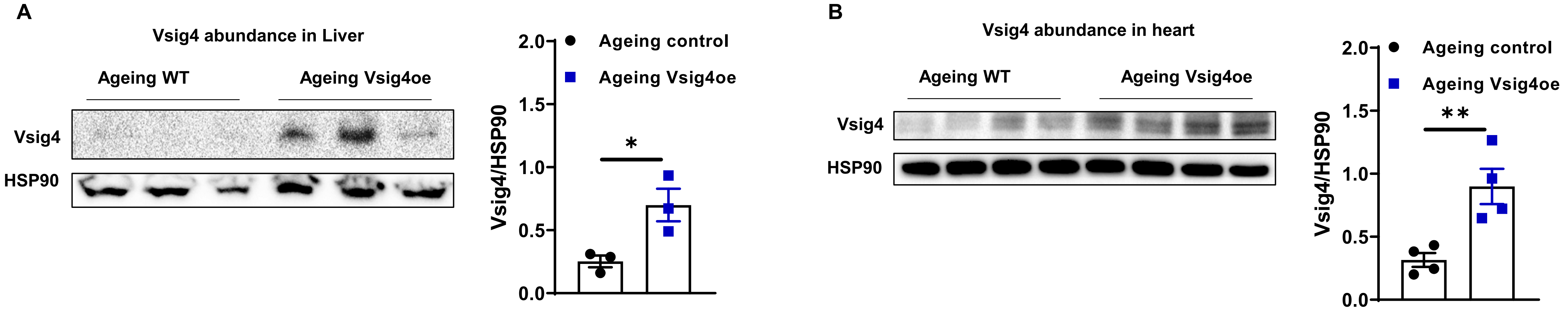

Figure S6. Visg4 expression in the livers and hearts of ageing WT mice after 2 weeks of injection with lentivirus carrying deactivated Cas9/VPR (VP64, p65, and Rta) and gRNA-Vsig4 TSS (transcriptional start site). Data are presented as mean ± SEM. *P* values are determined by Student's *t*-test.
