# Supplementary material for: Gut lumen-leaked microbial DNA causes myocardial inflammation and impairs cardiac contractility in ageing mouse heart
Source: Front Immunol. 2023 Jul 13;14:1216344. doi: 10.3389/fimmu.2023.1216344 (PMC10373503; doi:10.3389/fimmu.2023.1216344)
Supplement: Supplementary file 2 [file Table_1.pdf]

Table S1. Taxonomy summary of bacteria in ageing hearts and ileum

#OTU ID	Ageing.ileum.feces.1	Ageing.ileum.feces.2	Ageing.ileum.feces.3	Ageing.ileum.feces.4	Ageing.ileum.feces.5	Ageing.ileum.feces.6	Ageing.heart.1	Ageing.heart.2	Ageing.heart.3	Ageing.heart.4	Ageing.heart.5	Ageing.heart.6
k__Bacteria;p__Actinobacteria;c__Actinobacteria;o__Micrococcales;f__Dermabacteraceae;g__Brachybacterium;s__conglomeratum-faecium-paraconglomeratum	0	0	0	0	0	0.002042484	0	0	0	0	0	0
k__Bacteria;p__Actinobacteria;c__Actinobacteria;o__Micrococcales;f__Microbacteriaceae;g__Microbacterium;s__foliorum	0	0	0	0	0	0.003131808	0	0	0	0	0	0
k__Bacteria;p__Actinobacteria;c__Actinobacteria;o__Micrococcales;f__Micrococcaceae;g__Kocuria;s__assamensis-palustris	0	0	0	0	0	0.001293573	0	0	0	0	0	0
k__Bacteria;p__Actinobacteria;c__Actinobacteria;o__Micrococcales;f__Micrococcaceae;g__Rothia;s__amarae	0	0	0	0	0	0	0	0.000499667	0	0	0	0
k__Bacteria;p__Actinobacteria;c__Coriobacteriia;o__Coriobacteriales;f__Coriobacteriaceae;g__Adlercreutzia-Asaccharobacter;s__celatus-equolifaciens	0.001856472	0.00225331	0.002899065	0	0	0.001770153	0	0	0	0	0	0
k__Bacteria;p__Actinobacteria;c__Coriobacteriia;o__Coriobacteriales;f__Coriobacteriaceae;g__Enterorhabdus;s__caecimuris	0.024076115	0.036522392	0.039048633	0.002115686	0.003477563	0.010484749	0	0	0	0	0	0
k__Bacteria;p__Actinobacteria;c__Coriobacteriia;o__Coriobacteriales;f__Coriobacteriaceae;g__Enterorhabdus;s__mucosicola	0.00440912	0.007886583	0.011359602	0.005331528	0.002741925	0.021309913	0	0	0	0	0	0
k__Bacteria;p__Actinobacteria;c__Coriobacteriia;o__Coriobacteriales;f__Coriobacteriaceae;g__Enterorhabdus;s__sp10448	0.008934269	0.01427096	0.010590463	0.002031058	0.005015716	0.01123366	0	0	0.001611703	0	0	0
k__Bacteria;p__Actinobacteria;c__Coriobacteriia;o__Coriobacteriales;f__Coriobacteriaceae;g__Enterorhabdus;s__sp10449	0	0	0	0.000888588	0	0.004493464	0	0	0	0	0	0
k__Bacteria;p__Actinobacteria;c__Coriobacteriia;o__Coriobacteriales;f__Coriobacteriaceae;g__NA;s__sp10535	0.001856472	0.002534973	0.001597444	0.000973215	0	0.002382898	0	0	0	0	0	0
k__Bacteria;p__Actinobacteria;c__Coriobacteriia;o__Coriobacteriales;f__Coriobacteriaceae;g__Parvibacter;s__caecicola	0	0.001032767	0	0.003554352	0.0014044	0.010620915	0	0	0	0	0	0
k__Bacteria;p__Actinobacteria;c__Coriobacteriia;o__Coriobacteriales;f__Coriobacteriaceae;g__Senegalimassilia;s__sp10471	0.000754192	0.002910525	0.001301621	0	0	0	0	0	0	0	0	0
k__Bacteria;p__Bacteroidetes;c__Bacteroidia;o__Bacteroidales;f__NA;g__NA;s__sp12475-sp12557	0.098276962	0.10984884	0.062063661	0.004019803	0.005416973	0.005787037	0	0	0	0	0	0
k__Bacteria;p__Bacteroidetes;c__Bacteroidia;o__Bacteroidales;f__NA;g__NA;s__sp12497	0.00330684	0.00356774	0.014909478	0	0	0	0	0	0	0	0	0
k__Bacteria;p__Bacteroidetes;c__Bacteroidia;o__Bacteroidales;f__NA;g__NA;s__sp12502	0.018158612	0.017838701	0.022068394	0	0	0	0	0	0	0	0	0
k__Bacteria;p__Bacteroidetes;c__Bacteroidia;o__Bacteroidales;f__NA;g__NA;s__sp12503	0	0	0.004141522	0	0	0	0	0	0	0	0	0
k__Bacteria;p__Bacteroidetes;c__Bacteroidia;o__Bacteroidales;f__NA;g__NA;s__sp12505	0	0	0	0.007574155	0.012305223	0.02171841	0	0	0	0	0	0
k__Bacteria;p__Bacteroidetes;c__Bacteroidia;o__Bacteroidales;f__NA;g__NA;s__sp12510	0	0	0	0	0	0	0.006384751	0.001374084	0.002603521	0	0	0
k__Bacteria;p__Bacteroidetes;c__Bacteroidia;o__Bacteroidales;f__NA;g__NA;s__sp12514	0.007541916	0.007698808	0.021950065	0.026107561	0.006687621	0.014978214	0.010548718	0.000832778	0	0	0	0
k__Bacteria;p__Bacteroidetes;c__Bacteroidia;o__Bacteroidales;f__NA;g__NA;s__sp12520	0.001624413	0.002628861	0.000887469	0	0	0	0	0	0	0	0	0
k__Bacteria;p__Bacteroidetes;c__Bacteroidia;o__Bacteroidales;f__NA;g__NA;s__sp12531-sp12733	0	0	0	0.016417721	0	0.011097495	0	0	0	0	0	0
k__Bacteria;p__Bacteroidetes;c__Bacteroidia;o__Bacteroidales;f__NA;g__NA;s__sp12532	0.003016766	0.003849404	0.007158916	0	0	0	0	0	0	0	0	0
k__Bacteria;p__Bacteroidetes;c__Bacteroidia;o__Bacteroidales;f__NA;g__NA;s__sp12539	0	0	0	0.010282232	0.028689895	0.015590959	0	0	0	0	0	0
k__Bacteria;p__Bacteroidetes;c__Bacteroidia;o__Bacteroidales;f__NA;g__NA;s__sp12543	0.016070082	0.013895409	0.004082357	0.005458469	0.00441383	0.011029412	0	0	0	0	0	0
k__Bacteria;p__Bacteroidetes;c__Bacteroidia;o__Bacteroidales;f__NA;g__NA;s__sp12550	0.047107965	0.049197258	0.019228494	0.023484111	0.028221762	0.021990741	0.001573055	0.000624584	0	0	0	0
k__Bacteria;p__Bacteroidetes;c__Bacteroidia;o__Bacteroidales;f__NA;g__NA;s__sp12565	0.005627429	0.009482678	0.008578866	0.003046587	0.003276934	0.006604031	0	0	0	0	0	0
k__Bacteria;p__Bacteroidetes;c__Bacteroidia;o__Bacteroidales;f__NA;g__NA;s__sp12590	0.002842722	0.001408318	0.003372382	0	0	0	0	0	0	0	0	0
k__Bacteria;p__Bacteroidetes;c__Bacteroidia;o__Bacteroidales;f__NA;g__NA;s__sp12610	0.038289726	0.04065346	0.02703822	0.031058266	0.023072293	0.021922658	0	0	0	0	0	0
k__Bacteria;p__Bacteroidetes;c__Bacteroidia;o__Bacteroidales;f__NA;g__NA;s__sp12623	0.025932587	0.027321378	0.012069578	0	0	0	0	0	0	0	0	0
k__Bacteria;p__Bacteroidetes;c__Bacteroidia;o__Bacteroidales;f__NA;g__NA;s__sp12631	0	0	0	0.003681293	0.003410687	0.002791394	0	0	0	0	0	0
k__Bacteria;p__Bacteroidetes;c__Bacteroidia;o__Bacteroidales;f__NA;g__NA;s__sp12641	0.011080815	0.006572153	0.051059046	0.070536961	0.093359192	0.065699891	0	0	0	0	0	0
k__Bacteria;p__Bacteroidetes;c__Bacteroidia;o__Bacteroidales;f__NA;g__NA;s__sp12645	0	0	0	0	0	0	0.002961044	0	0	0	0	0
k__Bacteria;p__Bacteroidetes;c__Bacteroidia;o__Bacteroidales;f__NA;g__NA;s__sp12647	0	0	0	0.011551644	0.028823647	0.008510349	0	0	0	0	0	0
k__Bacteria;p__Bacteroidetes;c__Bacteroidia;o__Bacteroidales;f__NA;g__NA;s__sp12656	0.011486918	0.0147404	0.007277245	0.016967799	0.032033706	0.045819717	0	0	0	0	0	0
k__Bacteria;p__Bacteroidetes;c__Bacteroidia;o__Bacteroidales;f__NA;g__NA;s__sp12666	0.005859488	0.005069946	0.008046385	0	0	0	0	0	0	0	0	0
k__Bacteria;p__Bacteroidetes;c__Bacteroidia;o__Bacteroidales;f__NA;g__NA;s__sp12674	0	0	0	0	0	0.001770153	0	0	0	0	0	0
k__Bacteria;p__Bacteroidetes;c__Bacteroidia;o__Bacteroidales;f__NA;g__NA;s__sp12684	0.027266926	0.042155666	0.021003432	0.002496509	0.015515281	0.010757081	0	0	0	0	0	0
k__Bacteria;p__Bacteroidetes;c__Bacteroidia;o__Bacteroidales;f__NA;g__NA;s__sp12690-sp12760	0	0	0	0	0.001538153	0	0	0	0	0	0	0
k__Bacteria;p__Bacteroidetes;c__Bacteroidia;o__Bacteroidales;f__NA;g__NA;s__sp12744	0	0	0	0.000465451	0.013575871	0.003404139	0	0	0	0	0	0
k__Bacteria;p__Bacteroidetes;c__Bacteroidia;o__Bacteroidales;f__NA;g__NA;s__sp12760	0	0	0	0	0	0.001633987	0	0	0	0	0	0
k__Bacteria;p__Bacteroidetes;c__Bacteroidia;o__Bacteroidales;f__NA;g__NA;s__sp12778	0	0	0	0.003554352	0.002073163	0.003880719	0	0	0	0	0	0
k__Bacteria;p__Bacteroidetes;c__Bacteroidia;o__Bacteroidales;f__NA;g__NA;s__sp12804	0.057550618	0.103933903	0.075079872	0	0	0	0	0	0	0	0	0
k__Bacteria;p__Cyanobacteria;c__Chloroplast;o__'Chlorella' mirabilis;f__'Chlorella' mirabilis;g__NA;s__sp21729-sp22790	0	0	0	0	0	0	0	0	0.001859658	0	0	0
k__Bacteria;p__Cyanobacteria;c__Chloroplast;o__Glycine soja;f__Glycine soja;g__Glycine;s__soja	0	0	0	0.000761647	0	0	0	0	0	0	0	0
k__Bacteria;p__Firmicutes;c__Bacilli;o__Bacillales;f__Staphylococcaceae;g__Jeotgalicoccus;s__halophilus-halotolerans-nanhaiensis	0	0	0	0.000465451	0	0.002519063	0	0	0	0	0	0
k__Bacteria;p__Firmicutes;c__Bacilli;o__Bacillales;f__Staphylococcaceae;g__Staphylococcus;s__cohnii-nepalensis	0	0	0	0.000465451	0	0.007284858	0	0.000458028	0	0	0	0
k__Bacteria;p__Firmicutes;c__Bacilli;o__Bacillales;f__Staphylococcaceae;g__Staphylococcus;s__saprophyticus-xylosus	0	0	0	0	0.000936267	0.001565904	0.265291015	0.004705197	0.008182494	0	0	0
k__Bacteria;p__Firmicutes;c__Bacilli;o__Lactobacillales;f__Aerococcaceae;g__Aerococcus;s__urinaeequi-viridans	0	0	0	0	0	0.005718954	0	0	0	0	0	0
k__Bacteria;p__Firmicutes;c__Bacilli;o__Lactobacillales;f__Carnobacteriaceae;g__Atopostipes;s__sp28157-suicloacalis	0	0	0	0	0	0.002587146	0	0	0	0	0	0
k__Bacteria;p__Firmicutes;c__Bacilli;o__Lactobacillales;f__Enterococcaceae;g__Enterococcus;s__casseliflavus-flavescens-gallinarum	0	0	0	0	0.001337524	0.001293573	0	0	0	0	0	0
k__Bacteria;p__Firmicutes;c__Bacilli;o__Lactobacillales;f__Enterococcaceae;g__Enterococcus;s__faecalis	0	0	0	0	0	0	0	0.938416056	0.789982643	0.024413146	0.089097304	0.046511628
k__Bacteria;p__Firmicutes;c__Bacilli;o__Lactobacillales;f__Enterococcaceae;g__Enterococcus;s__mundtii	0	0	0	0	0	0	0	0	0.003595338	0.017840376	0	0
k__Bacteria;p__Firmicutes;c__Bacilli;o__Lactobacillales;f__Lactobacillaceae;g__Lactobacillus;s__NA	0	0	0	0	0	0	0.432589988	0.001873751	0	0.876995305	0	0
k__Bacteria;p__Firmicutes;c__Bacilli;o__Lactobacillales;f__Lactobacillaceae;g__Lactobacillus;s__gasseri-hominis	0	0	0	0	0	0.000885076	0	0	0	0	0	0
k__Bacteria;p__Firmicutes;c__Bacilli;o__Lactobacillales;f__Lactobacillaceae;g__Lactobacillus;s__johnsonii	0.33689157	0.272932119	0.218258194	0.529090678	0.386210125	0.422453704	0.012121773	0.002789807	0.001983635	0.014084507	0	0
k__Bacteria;p__Firmicutes;c__Bacilli;o__Lactobacillales;f__Lactobacillaceae;g__Lactobacillus;s__reuteri-vaginalis	0.074432906	0.043563985	0.093006745	0.173993991	0.023072293	0.044662309	0	0.000791139	0	0	0	0
k__Bacteria;p__Firmicutes;c__Bacilli;o__Lactobacillales;f__Streptococcaceae;g__Streptococcus;s__salivarius-thermophilus	0	0	0	0.000507765	0	0.003744553	0	0	0	0	0	0
k__Bacteria;p__Firmicutes;c__Clostridia;o__Clostridiales;f__Defluviitaleaceae;g__NA;s__sp31136	0	0	0	0	0.001872534	0	0	0	0	0	0	0
k__Bacteria;p__Firmicutes;c__Clostridia;o__Clostridiales;f__Family XIII;g__NA;s__sp31564	0	0	0.001597444	0	0	0	0	0	0	0	0	0
k__Bacteria;p__Firmicutes;c__Clostridia;o__Clostridiales;f__Family XIII;g__NA;s__sp31622	0.000696177	0	0.001183292	0	0.003210058	0	0	0	0	0	0	0
k__Bacteria;p__Firmicutes;c__Clostridia;o__Clostridiales;f__Family XIII;g__NA;s__sp31651	0.000812206	0	0.001124127	0	0	0.000885076	0	0	0	0	0	0
k__Bacteria;p__Firmicutes;c__Clostridia;o__Clostridiales;f__Lachnospiraceae;g__Blautia;s__sp32009	0	0	0	0.001057843	0.005885107	0.000953159	0	0	0	0	0	0
k__Bacteria;p__Firmicutes;c__Clostridia;o__Clostridiales;f__Lachnospiraceae;g__Lachnoclostridium;s__sp32351	0.009630446	0.009201014	0.027097385	0	0	0	0	0	0	0	0	0
k__Bacteria;p__Firmicutes;c__Clostridia;o__Clostridiales;f__Lachnospiraceae;g__Lachnoclostridium;s__sp32387	0.000754192	0	0	0	0.001070019	0.001429739	0	0	0	0	0	0
k__Bacteria;p__Firmicutes;c__Clostridia;o__Clostridiales;f__Lachnospiraceae;g__Lachnoclostridium;s__sp32402	0	0	0	0	0	0.005174292	0	0	0	0	0	0
k__Bacteria;p__Firmicutes;c__Clostridia;o__Clostridiales;f__Lachnospiraceae;g__Lachnoclostridium;s__sp32442	0.013807507	0.008262135	0.017453556	0.017941015	0.07363071	0.052015251	0	0	0	0	0	0
k__Bacteria;p__Firmicutes;c__Clostridia;o__Clostridiales;f__Lachnospiraceae;g__Marvinbryantia;s__sp32979	0.013575448	0.012768754	0.028753994	0	0.001538153	0.009872004	0	0	0	0	0	0
k__Bacteria;p__Firmicutes;c__Clostridia;o__Clostridiales;f__Lachnospiraceae;g__NA;s__sp32166	0	0	0	0	0.001136896	0	0	0	0	0	0	0
k__Bacteria;p__Firmicutes;c__Clostridia;o__Clostridiales;f__Lachnospiraceae;g__NA;s__sp32254	0	0	0	0.001777176	0.005617602	0.004833878	0	0	0	0	0	0
k__Bacteria;p__Firmicutes;c__Clostridia;o__Clostridiales;f__Lachnospiraceae;g__NA;s__sp32255	0	0	0.001301621	0	0	0	0	0	0	0	0	0
k__Bacteria;p__Firmicutes;c__Clostridia;o__Clostridiales;f__Lachnospiraceae;g__NA;s__sp32256	0.001972501	0.004976059	0.003668205	0	0	0	0	0	0	0	0	0
k__Bacteria;p__Firmicutes;c__Clostridia;o__Clostridiales;f__Lachnospiraceae;g__NA;s__sp32270	0	0	0	0	0.002273791	0.000748911	0	0	0	0	0	0
k__Bacteria;p__Firmicutes;c__Clostridia;o__Clostridiales;f__Lachnospiraceae;g__NA;s__sp32498	0.000986251	0.001596094	0.00360904	0.000550078	0.002073163	0	0	0	0	0	0	0
k__Bacteria;p__Firmicutes;c__Clostridia;o__Clostridiales;f__Lachnospiraceae;g__NA;s__sp32510	0	0	0.001360786	0	0.001203772	0	0	0	0	0	0	0
k__Bacteria;p__Firmicutes;c__Clostridia;o__Clostridiales;f__Lachnospiraceae;g__NA;s__sp32614-sp32671	0	0	0	0	0.001805658	0.001565904	0	0	0	0	0	0
k__Bacteria;p__Firmicutes;c__Clostridia;o__Clostridiales;f__Lachnospiraceae;g__NA;s__sp32622	0	0	0	0	0	0.001974401	0	0	0	0	0	0
k__Bacteria;p__Firmicutes;c__Clostridia;o__Clostridiales;f__Lachnospiraceae;g__NA;s__sp32628-sp32767	0	0	0	0	0	0.000885076	0	0	0	0	0	0
k__Bacteria;p__Firmicutes;c__Clostridia;o__Clostridiales;f__Lachnospiraceae;g__NA;s__sp32630	0	0	0.000828304	0	0	0	0	0	0	0	0	0
k__Bacteria;p__Firmicutes;c__Clostridia;o__Clostridiales;f__Lachnospiraceae;g__NA;s__sp32668	0	0	0	0	0.00247442	0	0	0	0	0	0	0
k__Bacteria;p__Firmicutes;c__Clostridia;o__Clostridiales;f__Lachnospiraceae;g__NA;s__sp32701	0	0	0	0	0.001270648	0.006808279	0	0	0	0	0	0
k__Bacteria;p__Firmicutes;c__Clostridia;o__Clostridiales;f__Lachnospiraceae;g__NA;s__sp32704	0	0.00178387	0.000828304	0	0	0.007489107	0	0	0	0	0	0
k__Bacteria;p__Firmicutes;c__Clostridia;o__Clostridiales;f__Lachnospiraceae;g__NA;s__sp32719-sp32755	0	0	0	0	0.001805658	0	0	0	0	0	0	0
k__Bacteria;p__Firmicutes;c__Clostridia;o__Clostridiales;f__Lachnospiraceae;g__NA;s__sp32761	0	0	0	0	0	0.000816993	0	0	0	0	0	0
k__Bacteria;p__Firmicutes;c__Clostridia;o__Clostridiales;f__Lachnospiraceae;g__NA;s__sp32763	0	0	0	0	0.004012573	0.002859477	0	0	0	0	0	0
k__Bacteria;p__Firmicutes;c__Clostridia;o__Clostridiales;f__Lachnospiraceae;g__NA;s__sp32766	0	0.001314431	0	0	0	0.006467865	0	0	0	0	0	0
k__Bacteria;p__Firmicutes;c__Clostridia;o__Clostridiales;f__Lachnospiraceae;g__NA;s__sp32778	0	0	0.000650811	0	0	0	0	0	0	0	0	0
k__Bacteria;p__Firmicutes;c__Clostridia;o__Clostridiales;f__Lachnospiraceae;g__NA;s__sp32791	0	0	0	0	0	0.001429739	0	0	0	0	0	0
k__Bacteria;p__Firmicutes;c__Clostridia;o__Clostridiales;f__Lachnospiraceae;g__NA;s__sp32816	0	0	0	0	0	0.002110566	0	0	0	0	0	0
k__Bacteria;p__Firmicutes;c__Clostridia;o__Clostridiales;f__Lachnospiraceae;g__NA;s__sp32819	0	0	0	0	0.001203772	0	0	0	0	0	0	0
k__Bacteria;p__Firmicutes;c__Clostridia;o__Clostridiales;f__Lachnospiraceae;g__NA;s__sp32843	0	0	0	0	0.002073163	0	0	0	0	0	0	0
k__Bacteria;p__Firmicutes;c__Clostridia;o__Clostridiales;f__Lachnospiraceae;g__NA;s__sp32856-sp32859	0	0	0	0	0	0.000953159	0	0	0	0	0	0
k__Bacteria;p__Firmicutes;c__Clostridia;o__Clostridiales;f__Lachnospiraceae;g__NA;s__sp32862	0.002378604	0.001126655	0.003372382	0.00114247	0.009429546	0.000885076	0	0	0	0	0	0
k__Bacteria;p__Firmicutes;c__Clostridia;o__Clostridiales;f__Lachnospiraceae;g__NA;s__sp32872	0	0	0	0	0.005951983	0.000953159	0	0	0	0	0	0
k__Bacteria;p__Firmicutes;c__Clostridia;o__Clostridiales;f__Lachnospiraceae;g__NA;s__sp32883	0.003074781	0.002347197	0.006212283	0	0	0	0	0	0	0	0	0
k__Bacteria;p__Firmicutes;c__Clostridia;o__Clostridiales;f__Lachnospiraceae;g__NA;s__sp32885	0	0	0	0	0.004280078	0	0	0	0	0	0	0
k__Bacteria;p__Firmicutes;c__Clostridia;o__Clostridiales;f__Lachnospiraceae;g__NA;s__sp33416-sp33419	0.002378604	0.002534973	0.000650811	0	0	0.001293573	0	0	0	0	0	0
k__Bacteria;p__Firmicutes;c__Clostridia;o__Clostridiales;f__Lachnospiraceae;g__NA;s__sp33426	0.001276324	0	0	0	0.001203772	0.000953159	0	0	0	0	0	0
k__Bacteria;p__Firmicutes;c__Clostridia;o__Clostridiales;f__Lachnospiraceae;g__NA;s__sp33436	0	0	0	0	0.000735638	0	0	0	0	0	0	0
k__Bacteria;p__Firmicutes;c__Clostridia;o__Clostridiales;f__Lachnospiraceae;g__NA;s__sp33459	0	0	0	0	0	0.000816993	0	0	0	0	0	0
k__Bacteria;p__Firmicutes;c__Clostridia;o__Clostridiales;f__Lachnospiraceae;g__NA;s__sp33470	0.006265591	0.003943292	0.009466335	0.003427411	0.011368956	0.00708061	0	0	0.001487726	0	0	0
k__Bacteria;p__Firmicutes;c__Clostridia;o__Clostridiales;f__Lachnospiraceae;g__NA;s__sp33470-sp33680	0	0	0.002839901	0	0	0	0	0	0	0	0	0
k__Bacteria;p__Firmicutes;c__Clostridia;o__Clostridiales;f__Lachnospiraceae;g__NA;s__sp33503	0.001392354	0.00178387	0	0	0	0.003676471	0	0	0	0	0	0
k__Bacteria;p__Firmicutes;c__Clostridia;o__Clostridiales;f__Lachnospiraceae;g__NA;s__sp33513	0	0	0	0	0.001805658	0	0	0	0	0	0	0
k__Bacteria;p__Firmicutes;c__Clostridia;o__Clostridiales;f__Lachnospiraceae;g__NA;s__sp33518	0	0.001596094	0.001656609	0.001734862	0.007022002	0.001497821	0.003793837	0.000582945	0	0	0	0
k__Bacteria;p__Firmicutes;c__Clostridia;o__Clostridiales;f__Lachnospiraceae;g__NA;s__sp33529	0.000928236	0.001314431	0.005028991	0	0	0	0	0	0	0	0	0
k__Bacteria;p__Firmicutes;c__Clostridia;o__Clostridiales;f__Lachnospiraceae;g__NA;s__sp33539	0	0	0	0	0.001136896	0.001429739	0	0	0	0	0	0
k__Bacteria;p__Firmicutes;c__Clostridia;o__Clostridiales;f__Lachnospiraceae;g__NA;s__sp33540	0	0	0.001242457	0	0	0	0	0	0	0	0	0
k__Bacteria;p__Firmicutes;c__Clostridia;o__Clostridiales;f__Lachnospiraceae;g__NA;s__sp33558	0	0	0	0.001438666	0.005483849	0	0	0	0	0	0	0
k__Bacteria;p__Firmicutes;c__Clostridia;o__Clostridiales;f__Lachnospiraceae;g__NA;s__sp33565	0	0	0.005620637	0	0	0	0	0	0	0	0	0
k__Bacteria;p__Firmicutes;c__Clostridia;o__Clostridiales;f__Lachnospiraceae;g__NA;s__sp33582	0.004061032	0.003755516	0.003313217	0.002538823	0.006286364	0.002110566	0	0	0	0	0	0
k__Bacteria;p__Firmicutes;c__Clostridia;o__Clostridiales;f__Lachnospiraceae;g__NA;s__sp33589	0	0	0	0	0.001270648	0	0	0	0	0	0	0
k__Bacteria;p__Firmicutes;c__Clostridia;o__Clostridiales;f__Lachnospiraceae;g__NA;s__sp33604	0.018100598	0.021030889	0.028635664	0	0	0	0	0	0	0	0	0
k__Bacteria;p__Firmicutes;c__Clostridia;o__Clostridiales;f__Lachnospiraceae;g__NA;s__sp33616-sp33639	0.00539537	0.007041592	0.022600876	0.002581137	0	0.000953159	0	0	0	0	0	0
k__Bacteria;p__Firmicutes;c__Clostridia;o__Clostridiales;f__Lachnospiraceae;g__NA;s__sp33636	0	0	0	0.000592392	0.01324149	0.00292756	0	0	0	0	0	0
k__Bacteria;p__Firmicutes;c__Clostridia;o__Clostridiales;f__Lachnospiraceae;g__NA;s__sp33639	0.010210593	0.006008825	0.009407171	0	0.021199759	0.003472222	0	0	0	0	0	0
k__Bacteria;p__Firmicutes;c__Clostridia;o__Clostridiales;f__Lachnospiraceae;g__NA;s__sp33658	0.001624413	0.001502206	0.00295823	0	0	0.001089325	0	0	0	0	0	0
k__Bacteria;p__Firmicutes;c__Clostridia;o__Clostridiales;f__Lachnospiraceae;g__NA;s__sp33670	0.002610663	0.002628861	0.001183292	0	0.001070019	0.001497821	0	0	0	0	0	0
k__Bacteria;p__Firmicutes;c__Clostridia;o__Clostridiales;f__Lachnospiraceae;g__NA;s__sp33674	0.006671695	0.009294902	0.007395574	0	0	0	0	0	0	0	0	0
k__Bacteria;p__Firmicutes;c__Clostridia;o__Clostridiales;f__Lachnospiraceae;g__NA;s__sp33694	0	0	0	0.000507765	0.002340667	0	0	0	0	0	0	0
k__Bacteria;p__Firmicutes;c__Clostridia;o__Clostridiales;f__Lachnospiraceae;g__NA;s__sp33734	0	0	0	0.000677019	0	0	0	0	0	0	0	0
k__Bacteria;p__Firmicutes;c__Clostridia;o__Clostridiales;f__Lachnospiraceae;g__NA;s__sp33738	0	0.001314431	0.008283043	0.002157999	0.003678192	0	0.001110391	0	0	0	0	0
k__Bacteria;p__Firmicutes;c__Clostridia;o__Clostridiales;f__Lachnospiraceae;g__NA;s__sp33760	0	0.001032767	0	0	0.000936267	0	0	0	0	0	0	0
k__Bacteria;p__Firmicutes;c__Clostridia;o__Clostridiales;f__Lachnospiraceae;g__NA;s__sp33761	0	0	0.001479115	0	0.006955126	0	0	0	0	0	0	0
k__Bacteria;p__Firmicutes;c__Clostridia;o__Clostridiales;f__Lachnospiraceae;g__NA;s__sp33766	0.001276324	0.001971646	0	0	0.002273791	0.000748911	0	0	0	0	0	0
k__Bacteria;p__Firmicutes;c__Clostridia;o__Clostridiales;f__Lachnospiraceae;g__NA;s__sp33767	0	0	0	0	0.002541296	0.000748911	0	0	0	0	0	0
k__Bacteria;p__Firmicutes;c__Clostridia;o__Clostridiales;f__Lachnospiraceae;g__Roseburia;s__sp33136	0.000638162	0	0.00076914	0	0	0	0	0	0	0	0	0
k__Bacteria;p__Firmicutes;c__Clostridia;o__Clostridiales;f__Lachnospiraceae;g__Roseburia;s__sp33136-sp33149	0	0	0	0	0.000735638	0	0	0	0	0	0	0
k__Bacteria;p__Firmicutes;c__Clostridia;o__Clostridiales;f__Lachnospiraceae;g__Roseburia;s__sp33140	0	0	0.00076914	0	0	0	0	0	0	0	0	0
k__Bacteria;p__Firmicutes;c__Clostridia;o__Clostridiales;f__Lachnospiraceae;g__Roseburia;s__sp33143	0.001914486	0.004318843	0.002780736	0	0	0	0	0	0	0	0	0
k__Bacteria;p__Firmicutes;c__Clostridia;o__Clostridiales;f__Lachnospiraceae;g__Roseburia;s__sp33149	0	0	0	0.000846274	0.001805658	0	0	0	0	0	0	0
k__Bacteria;p__Firmicutes;c__Clostridia;o__Clostridiales;f__Lachnospiraceae;g__Roseburia;s__sp33152	0	0	0	0	0	0.00245098	0	0	0	0	0	0
k__Bacteria;p__Firmicutes;c__Clostridia;o__Clostridiales;f__Lachnospiraceae;g__Roseburia;s__sp33168	0	0.001220543	0	0	0.003544439	0	0	0	0	0	0	0
k__Bacteria;p__Firmicutes;c__Clostridia;o__Clostridiales;f__Lachnospiraceae;g__Roseburia;s__sp33177	0	0	0	0	0.000735638	0	0	0	0	0	0	0
k__Bacteria;p__Firmicutes;c__Clostridia;o__Clostridiales;f__NA;g__*Arthromitus;s__sp30492	0	0	0.008046385	0	0	0	0	0	0	0	0	0
k__Bacteria;p__Firmicutes;c__Clostridia;o__Clostridiales;f__Peptostreptococcaceae;g__Terrisporobacter;s__sp34393	0	0	0	0	0	0.000748911	0	0	0	0	0	0
k__Bacteria;p__Firmicutes;c__Clostridia;o__Clostridiales;f__Ruminococcaceae;g__NA;s__sp34764	0.000812206	0	0	0	0	0	0	0	0	0	0	0
k__Bacteria;p__Firmicutes;c__Clostridia;o__Clostridiales;f__Ruminococcaceae;g__NA;s__sp34771	0.001044265	0.001032767	0.005265649	0	0	0	0	0	0	0	0	0
k__Bacteria;p__Firmicutes;c__Clostridia;o__Clostridiales;f__Ruminococcaceae;g__NA;s__sp34795-sp34820	0	0	0.004200686	0	0	0	0	0	0	0	0	0
k__Bacteria;p__Firmicutes;c__Clostridia;o__Clostridiales;f__Ruminococcaceae;g__NA;s__sp35297	0.002726693	0.001126655	0.001479115	0	0.00581823	0	0	0	0	0	0	0
k__Bacteria;p__Firmicutes;c__Clostridia;o__Clostridiales;f__Ruminococcaceae;g__NA;s__sp35328-sp35341-sp35377	0	0	0	0	0.002407544	0	0	0	0	0	0	0
k__Bacteria;p__Firmicutes;c__Clostridia;o__Clostridiales;f__Ruminococcaceae;g__NA;s__sp35341	0	0	0	0	0.001671905	0.003063725	0	0	0	0	0	0
k__Bacteria;p__Firmicutes;c__Clostridia;o__Clostridiales;f__Ruminococcaceae;g__NA;s__sp35351	0	0	0	0	0.00300943	0	0	0	0	0	0	0
k__Bacteria;p__Firmicutes;c__Clostridia;o__Clostridiales;f__Ruminococcaceae;g__NA;s__sp35382	0	0	0	0	0.005951983	0.001429739	0	0	0	0	0	0
k__Bacteria;p__Firmicutes;c__Clostridia;o__Clostridiales;f__Ruminococcaceae;g__NA;s__sp35382-sp35403-sp35432	0	0	0	0	0.002808801	0	0	0	0	0	0	0
k__Bacteria;p__Firmicutes;c__Clostridia;o__Clostridiales;f__Ruminococcaceae;g__NA;s__sp35403	0.001624413	0.001032767	0.002721571	0	0	0	0	0	0	0	0	0
k__Bacteria;p__Firmicutes;c__Clostridia;o__Clostridiales;f__Ruminococcaceae;g__NA;s__sp35424	0	0	0	0.001607921	0.009496422	0	0.001665587	0	0	0	0	0
k__Bacteria;p__Firmicutes;c__Clostridia;o__Clostridiales;f__Ruminococcaceae;g__NA;s__sp35432	0	0	0.00141995	0	0	0	0	0	0	0	0	0
k__Bacteria;p__Firmicutes;c__Clostridia;o__Clostridiales;f__Ruminococcaceae;g__NA;s__sp35498	0	0	0.00076914	0	0.000735638	0	0	0	0	0	0	0
k__Bacteria;p__Firmicutes;c__Clostridia;o__Clostridiales;f__Ruminococcaceae;g__NA;s__sp35597-sp35598	0	0	0	0	0.001671905	0	0	0	0	0	0	0
k__Bacteria;p__Firmicutes;c__Clostridia;o__Clostridiales;f__Ruminococcaceae;g__NA;s__sp35598	0.001682427	0.001877758	0.002366584	0	0	0	0	0	0	0	0	0
k__Bacteria;p__Firmicutes;c__Clostridia;o__Clostridiales;f__Ruminococcaceae;g__NA;s__sp35736	0.000638162	0.001126655	0.002425748	0.000592392	0.002006286	0.001021242	0	0	0	0	0	0
k__Bacteria;p__Firmicutes;c__Clostridia;o__Clostridiales;f__Ruminococcaceae;g__NA;s__sp35799	0	0	0	0	0	0.001293573	0	0	0	0	0	0
k__Bacteria;p__Firmicutes;c__Clostridia;o__Clostridiales;f__Ruminococcaceae;g__NA;s__sp35832-sp35839	0	0	0	0	0.001337524	0	0	0	0	0	0	0
k__Bacteria;p__Firmicutes;c__Clostridia;o__Clostridiales;f__Ruminococcaceae;g__Oscillibacter;s__sp34650-sp34654	0	0	0	0	0.000802515	0	0	0	0	0	0	0
k__Bacteria;p__Firmicutes;c__Clostridia;o__Clostridiales;f__Ruminococcaceae;g__Ruminiclostridium;s__sp34925	0.002900737	0.003286076	0	0	0	0.000748911	0	0	0	0	0	0
k__Bacteria;p__Firmicutes;c__Clostridia;o__Clostridiales;f__Ruminococcaceae;g__Ruminococcus;s__bromii	0	0	0	0	0.001605029	0	0	0	0	0	0	0
k__Bacteria;p__Firmicutes;c__Erysipelotrichia;o__Erysipelotrichales;f__Erysipelotrichaceae;g__Allobaculum;s__sp36555	0	0	0	0.000846274	0	0.003267974	0	0	0	0	0	0
k__Bacteria;p__Firmicutes;c__Erysipelotrichia;o__Erysipelotrichales;f__Erysipelotrichaceae;g__Erysipelatoclostridium;s__sp36616-sp36629	0.005627429	0.005914938	0.003313217	0	0	0.002042484	0	0	0	0	0	0
k__Bacteria;p__Firmicutes;c__Erysipelotrichia;o__Erysipelotrichales;f__Erysipelotrichaceae;g__NA;s__sp36783	0.001334339	0	0.000828304	0	0.001136896	0.001770153	0	0	0	0	0	0
k__Bacteria;p__Firmicutes;c__Erysipelotrichia;o__Erysipelotrichales;f__Erysipelotrichaceae;g__NA;s__sp36786	0.00220456	0.00225331	0.00076914	0	0.000802515	0	0	0	0	0	0	0
k__Bacteria;p__Firmicutes;c__Erysipelotrichia;o__Erysipelotrichales;f__Erysipelotrichaceae;g__NA;s__sp36793	0.001160295	0	0	0	0	0	0	0	0	0	0	0
k__Bacteria;p__Proteobacteria;c__Alphaproteobacteria;o__Rhizobiales;f__Methylocystaceae;g__Methylocystis;s__Methylocystis-echinoides-parvus	0	0	0	0	0	0.000885076	0	0	0	0	0	0
k__Bacteria;p__Proteobacteria;c__Alphaproteobacteria;o__Rhodobacterales;f__Rhodobacteraceae;g__Paracoccus;s__sp44877	0	0	0	0.000634706	0	0.00170207	0	0	0	0	0	0
k__Bacteria;p__Proteobacteria;c__Alphaproteobacteria;o__Rhodospirillales;f__Acetobacteraceae;g__Craurococcus;s__sp45574-sp45578	0	0	0	0	0	0	0	0	0.001487726	0	0	0
k__Bacteria;p__Proteobacteria;c__Alphaproteobacteria;o__Rickettsiales;f__Mitochondria;g__Arundo-Oryza meyeriana;s__NA	0	0	0	0	0	0.000748911	0	0	0	0	0	0
k__Bacteria;p__Proteobacteria;c__Betaproteobacteria;o__Burkholderiales;f__Burkholderiaceae;g__Burkholderia-Paraburkholderia;s__insulsa	0	0	0	0	0	0	0.001017859	0	0	0	0	0
k__Bacteria;p__Proteobacteria;c__Betaproteobacteria;o__Burkholderiales;f__Comamonadaceae;g__Aquabacterium;s__parvum	0	0	0	0	0	0	0.006199685	0	0	0	0	0
k__Bacteria;p__Proteobacteria;c__Gammaproteobacteria;o__Pseudomonadales;f__Moraxellaceae;g__Acinetobacter;s__lwoffii	0	0	0	0	0	0	0.128620339	0.045594604	0.025787255	0	0	0
k__Bacteria;p__Proteobacteria;c__Gammaproteobacteria;o__Pseudomonadales;f__Moraxellaceae;g__Enhydrobacter-Moraxella;s__aerosaccus-osloensis	0	0	0	0	0	0	0	0	0.001487726	0	0	0
k__Bacteria;p__Proteobacteria;c__Gammaproteobacteria;o__Pseudomonadales;f__Pseudomonadaceae;g__Pseudomonas;s__aeruginosa	0	0	0	0	0	0	0.002683446	0	0.002479544	0	0	0
k__Bacteria;p__Tenericutes;c__Mollicutes;o__Anaeroplasmatales;f__Anaeroplasmataceae;g__Anaeroplasma;s__sp67615	0	0	0	0	0	0.000953159	0	0	0	0	0	0
k__Bacteria;p__Tenericutes;c__Mollicutes;o__NA;f__NA;g__NA;s__sp67846	0	0	0	0	0.000936267	0	0	0	0	0	0	0
k__Bacteria;p__Verrucomicrobia;c__Verrucomicrobiae;o__Verrucomicrobiales;f__Verrucomicrobiaceae;g__Akkermansia;s__muciniphila	0.031560016	0.021781992	0.003194888	0	0	0	0.00175812	0	0	0	0	0
None;Other;Other;Other;Other;Other;Other	0	0.001126655	0	0.000465451	0	0	0.121680392	0.001457362	0.157451029	0.066666667	0.910902696	0.953488372
