# Supplementary material for: Gut lumen-leaked microbial DNA causes myocardial inflammation and impairs cardiac contractility in ageing mouse heart
Source: Front Immunol. 2023 Jul 13;14:1216344. doi: 10.3389/fimmu.2023.1216344 (PMC10373503; doi:10.3389/fimmu.2023.1216344)
Supplement: Supplementary file 3 [file Table_2.pdf]

Table S2. Taxonomy summary of bacteria in young vs. ageing ileum

#OTU ID	Young.ileum.feces.1	Young.ileum.feces.2	Young.ileum.feces.3	Young.ileum.feces.4	Young.ileum.feces.5	Young.ileum.feces.6	Ageing.ileum.feces.1	Ageing.ileum.feces.2	Ageing.ileum.feces.3	Ageing.ileum.feces.4	Ageing.ileum.feces.5	Ageing.ileum.feces.6
k__Bacteria;p__Actinobacteria;c__Actinobacteria;o__Micrococcales;f__Dermabacteraceae;g__Brachybacterium;s__conglomeratum-faecium-paraconglomeratum	0	0	0	0	0	0	0	0	0	0	0	0.002042484
k__Bacteria;p__Actinobacteria;c__Actinobacteria;o__Micrococcales;f__Microbacteriaceae;g__Microbacterium;s__foliorum	0	0	0	0	0	0	0	0	0	0	0	0.003131808
k__Bacteria;p__Actinobacteria;c__Actinobacteria;o__Micrococcales;f__Micrococcaceae;g__Kocuria;s__assamensis-palustris	0	0	0	0	0	0	0	0	0	0	0	0.001293573
k__Bacteria;p__Actinobacteria;c__Coriobacteriia;o__Coriobacteriales;f__Coriobacteriaceae;g__Adlercreutzia-Asaccharobacter;s__celatus-equolifaciens	0.009377281	0.007689452	0.009151843	0.00505958	0.008072232	0.010711163	0.001856472	0.00225331	0.002899065	0	0	0.001770153
k__Bacteria;p__Actinobacteria;c__Coriobacteriia;o__Coriobacteriales;f__Coriobacteriaceae;g__Enterorhabdus;s__caecimuris	0	0	0	0	0	0	0.024076115	0.036522392	0.039048633	0.002115686	0.003477563	0.010484749
k__Bacteria;p__Actinobacteria;c__Coriobacteriia;o__Coriobacteriales;f__Coriobacteriaceae;g__Enterorhabdus;s__mucosicola	0.004772868	0.003393965	0.003738077	0.001229431	0.004783545	0.008525211	0.00440912	0.007886583	0.011359602	0.005331528	0.002741925	0.021309913
k__Bacteria;p__Actinobacteria;c__Coriobacteriia;o__Coriobacteriales;f__Coriobacteriaceae;g__Enterorhabdus;s__sp10448	0.002807569	0.000795461	0.001095643	0	0.001734035	0.002477412	0.008934269	0.01427096	0.010590463	0.002031058	0.005015716	0.01123366
k__Bacteria;p__Actinobacteria;c__Coriobacteriia;o__Coriobacteriales;f__Coriobacteriaceae;g__Enterorhabdus;s__sp10449	0	0	0	0	0	0	0	0	0	0.000888588	0	0.004493464
k__Bacteria;p__Actinobacteria;c__Coriobacteriia;o__Coriobacteriales;f__Coriobacteriaceae;g__NA;s__sp10535	0	0	0	0	0	0	0.001856472	0.002534973	0.001597444	0.000973215	0	0.002382898
k__Bacteria;p__Actinobacteria;c__Coriobacteriia;o__Coriobacteriales;f__Coriobacteriaceae;g__Parvibacter;s__caecicola	0	0	0	0	0	0.000801516	0	0.001032767	0	0.003554352	0.0014044	0.010620915
k__Bacteria;p__Actinobacteria;c__Coriobacteriia;o__Coriobacteriales;f__Coriobacteriaceae;g__Senegalimassilia;s__sp10471	0	0	0	0	0	0	0.000754192	0.002910525	0.001301621	0	0	0
k__Bacteria;p__Bacteroidetes;c__Bacteroidia;o__Bacteroidales;f__NA;g__NA;s__sp12475-sp12557	0	0	0	0	0	0	0.098276962	0.10984884	0.062063661	0.004019803	0.005416973	0.005787037
k__Bacteria;p__Bacteroidetes;c__Bacteroidia;o__Bacteroidales;f__NA;g__NA;s__sp12497	0	0	0	0	0	0	0.00330684	0.00356774	0.014909478	0	0	0
k__Bacteria;p__Bacteroidetes;c__Bacteroidia;o__Bacteroidales;f__NA;g__NA;s__sp12502	0	0	0	0	0	0	0.018158612	0.017838701	0.022068394	0	0	0
k__Bacteria;p__Bacteroidetes;c__Bacteroidia;o__Bacteroidales;f__NA;g__NA;s__sp12503	0	0	0	0	0	0	0	0	0.004141522	0	0	0
k__Bacteria;p__Bacteroidetes;c__Bacteroidia;o__Bacteroidales;f__NA;g__NA;s__sp12505	0	0	0	0	0	0	0	0	0	0.007574155	0.012305223	0.02171841
k__Bacteria;p__Bacteroidetes;c__Bacteroidia;o__Bacteroidales;f__NA;g__NA;s__sp12510	0.127070582	0.109349313	0.131219386	0.056837526	0.12215977	0.144272807	0	0	0	0	0	0
k__Bacteria;p__Bacteroidetes;c__Bacteroidia;o__Bacteroidales;f__NA;g__NA;s__sp12514	0.128867427	0.117993318	0.141724671	0.055277095	0.130530973	0.142888371	0.007541916	0.007698808	0.021950065	0.026107561	0.006687621	0.014978214
k__Bacteria;p__Bacteroidetes;c__Bacteroidia;o__Bacteroidales;f__NA;g__NA;s__sp12520	0	0	0	0	0	0	0.001624413	0.002628861	0.000887469	0	0	0
k__Bacteria;p__Bacteroidetes;c__Bacteroidia;o__Bacteroidales;f__NA;g__NA;s__sp12531-sp12733	0	0	0	0	0	0	0	0	0	0.016417721	0	0.011097495
k__Bacteria;p__Bacteroidetes;c__Bacteroidia;o__Bacteroidales;f__NA;g__NA;s__sp12532	0	0	0	0	0	0	0.003016766	0.003849404	0.007158916	0	0	0
k__Bacteria;p__Bacteroidetes;c__Bacteroidia;o__Bacteroidales;f__NA;g__NA;s__sp12539	0	0	0	0	0	0	0	0	0	0.010282232	0.028689895	0.015590959
k__Bacteria;p__Bacteroidetes;c__Bacteroidia;o__Bacteroidales;f__NA;g__NA;s__sp12543	0	0	0	0	0	0	0.016070082	0.013895409	0.004082357	0.005458469	0.00441383	0.011029412
k__Bacteria;p__Bacteroidetes;c__Bacteroidia;o__Bacteroidales;f__NA;g__NA;s__sp12550	0.100960189	0.092379488	0.119554009	0.035133346	0.095910069	0.106091519	0.047107965	0.049197258	0.019228494	0.023484111	0.028221762	0.021990741
k__Bacteria;p__Bacteroidetes;c__Bacteroidia;o__Bacteroidales;f__NA;g__NA;s__sp12565	0	0	0	0	0	0	0.005627429	0.009482678	0.008578866	0.003046587	0.003276934	0.006604031
k__Bacteria;p__Bacteroidetes;c__Bacteroidia;o__Bacteroidales;f__NA;g__NA;s__sp12590	0	0	0	0	0	0	0.002842722	0.001408318	0.003372382	0	0	0
k__Bacteria;p__Bacteroidetes;c__Bacteroidia;o__Bacteroidales;f__NA;g__NA;s__sp12610	0.024201247	0.020788036	0.023201856	0.009646302	0.028521885	0.027980181	0.038289726	0.04065346	0.02703822	0.031058266	0.023072293	0.021922658
k__Bacteria;p__Bacteroidetes;c__Bacteroidia;o__Bacteroidales;f__NA;g__NA;s__sp12623	0	0	0	0	0	0	0.025932587	0.027321378	0.012069578	0	0	0
k__Bacteria;p__Bacteroidetes;c__Bacteroidia;o__Bacteroidales;f__NA;g__NA;s__sp12631	0	0	0	0	0	0	0	0	0	0.003681293	0.003410687	0.002791394
k__Bacteria;p__Bacteroidetes;c__Bacteroidia;o__Bacteroidales;f__NA;g__NA;s__sp12641	0	0	0	0	0	0	0.011080815	0.006572153	0.051059046	0.070536961	0.093359192	0.065699891
k__Bacteria;p__Bacteroidetes;c__Bacteroidia;o__Bacteroidales;f__NA;g__NA;s__sp12645	0.047728677	0.037227555	0.036800722	0.020994893	0.032886869	0.048673856	0	0	0	0	0	0
k__Bacteria;p__Bacteroidetes;c__Bacteroidia;o__Bacteroidales;f__NA;g__NA;s__sp12647	0	0	0	0	0	0	0	0	0	0.011551644	0.028823647	0.008510349
k__Bacteria;p__Bacteroidetes;c__Bacteroidia;o__Bacteroidales;f__NA;g__NA;s__sp12656	0.008422708	0.005356101	0.00799175	0.002931719	0.010165032	0.015228796	0.011486918	0.0147404	0.007277245	0.016967799	0.032033706	0.045819717
k__Bacteria;p__Bacteroidetes;c__Bacteroidia;o__Bacteroidales;f__NA;g__NA;s__sp12666	0	0	0	0	0	0	0.005859488	0.005069946	0.008046385	0	0	0
k__Bacteria;p__Bacteroidetes;c__Bacteroidia;o__Bacteroidales;f__NA;g__NA;s__sp12674	0	0	0	0	0	0	0	0	0	0	0	0.001770153
k__Bacteria;p__Bacteroidetes;c__Bacteroidia;o__Bacteroidales;f__NA;g__NA;s__sp12684	0	0	0	0	0	0	0.027266926	0.042155666	0.021003432	0.002496509	0.015515281	0.010757081
k__Bacteria;p__Bacteroidetes;c__Bacteroidia;o__Bacteroidales;f__NA;g__NA;s__sp12690-sp12760	0	0	0	0	0	0	0	0	0	0	0.001538153	0
k__Bacteria;p__Bacteroidetes;c__Bacteroidia;o__Bacteroidales;f__NA;g__NA;s__sp12744	0	0	0	0	0	0	0	0	0	0.000465451	0.013575871	0.003404139
k__Bacteria;p__Bacteroidetes;c__Bacteroidia;o__Bacteroidales;f__NA;g__NA;s__sp12760	0	0	0	0	0	0	0	0	0	0	0	0.001633987
k__Bacteria;p__Bacteroidetes;c__Bacteroidia;o__Bacteroidales;f__NA;g__NA;s__sp12778	0	0	0	0	0	0	0	0	0	0.003554352	0.002073163	0.003880719
k__Bacteria;p__Bacteroidetes;c__Bacteroidia;o__Bacteroidales;f__NA;g__NA;s__sp12804	0	0	0	0	0	0	0.057550618	0.103933903	0.075079872	0	0	0
k__Bacteria;p__Bacteroidetes;c__Bacteroidia;o__Bacteroidales;f__NA;g__NA;s__sp12805	0.008198102	0.014848597	0.007347254	0.007329298	0.012855776	0.004299038	0	0	0	0	0	0
k__Bacteria;p__Cyanobacteria;c__Chloroplast;o__Glycine soja;f__Glycine soja;g__Glycine;s__soja	0	0	0	0	0	0	0	0	0	0.000761647	0	0
k__Bacteria;p__Firmicutes;c__Bacilli;o__Bacillales;f__Staphylococcaceae;g__Jeotgalicoccus;s__halophilus-halotolerans-nanhaiensis	0	0	0	0	0	0	0	0	0	0.000465451	0	0.002519063
k__Bacteria;p__Firmicutes;c__Bacilli;o__Bacillales;f__Staphylococcaceae;g__Staphylococcus;s__cohnii-nepalensis	0	0	0	0	0	0	0	0	0	0.000465451	0	0.007284858
k__Bacteria;p__Firmicutes;c__Bacilli;o__Bacillales;f__Staphylococcaceae;g__Staphylococcus;s__saprophyticus-xylosus	0	0.000583338	0	0	0	0	0	0	0	0	0.000936267	0.001565904
k__Bacteria;p__Firmicutes;c__Bacilli;o__Lactobacillales;f__Aerococcaceae;g__Aerococcus;s__urinaeequi-viridans	0	0	0	0	0	0	0	0	0	0	0	0.005718954
k__Bacteria;p__Firmicutes;c__Bacilli;o__Lactobacillales;f__Carnobacteriaceae;g__Atopostipes;s__sp28157-suicloacalis	0	0	0	0	0	0	0	0	0	0	0	0.002587146
k__Bacteria;p__Firmicutes;c__Bacilli;o__Lactobacillales;f__Enterococcaceae;g__Enterococcus;s__casseliflavus-flavescens-gallinarum	0	0	0	0	0	0	0	0	0	0	0.001337524	0.001293573
k__Bacteria;p__Firmicutes;c__Bacilli;o__Lactobacillales;f__Lactobacillaceae;g__Lactobacillus;s__NA	0.077152002	0.01956833	0.022750709	0.058303386	0.04275293	0.088531041	0	0	0	0	0	0
k__Bacteria;p__Firmicutes;c__Bacilli;o__Lactobacillales;f__Lactobacillaceae;g__Lactobacillus;s__gasseri-hominis	0	0	0	0	0	0	0	0	0	0	0	0.000885076
k__Bacteria;p__Firmicutes;c__Bacilli;o__Lactobacillales;f__Lactobacillaceae;g__Lactobacillus;s__johnsonii	0.269077433	0.493874954	0.344934261	0.693351617	0.367196843	0.273025357	0.33689157	0.272932119	0.218258194	0.529090678	0.386210125	0.422453704
k__Bacteria;p__Firmicutes;c__Bacilli;o__Lactobacillales;f__Lactobacillaceae;g__Lactobacillus;s__reuteri-vaginalis	0	0	0	0	0	0	0.074432906	0.043563985	0.093006745	0.173993991	0.023072293	0.044662309
k__Bacteria;p__Firmicutes;c__Bacilli;o__Lactobacillales;f__Streptococcaceae;g__Streptococcus;s__salivarius-thermophilus	0	0	0	0	0	0	0	0	0	0.000507765	0	0.003744553
k__Bacteria;p__Firmicutes;c__Clostridia;o__Clostridiales;f__Defluviitaleaceae;g__NA;s__sp31136	0	0	0	0	0	0	0	0	0	0	0.001872534	0
k__Bacteria;p__Firmicutes;c__Clostridia;o__Clostridiales;f__Family XIII;g__NA;s__sp31564	0	0	0	0	0	0	0	0	0.001597444	0	0	0
k__Bacteria;p__Firmicutes;c__Clostridia;o__Clostridiales;f__Family XIII;g__NA;s__sp31622	0	0	0.001160093	0	0	0	0.000696177	0	0.001183292	0	0.003210058	0
k__Bacteria;p__Firmicutes;c__Clostridia;o__Clostridiales;f__Family XIII;g__NA;s__sp31651	0	0	0	0	0	0	0.000812206	0	0.001124127	0	0	0.000885076
k__Bacteria;p__Firmicutes;c__Clostridia;o__Clostridiales;f__Lachnospiraceae;g__Blautia;s__sp32009	0	0	0	0	0	0.003570388	0	0	0	0.001057843	0.005885107	0.000953159
k__Bacteria;p__Firmicutes;c__Clostridia;o__Clostridiales;f__Lachnospiraceae;g__Blautia;s__sp32015	0.001010725	0	0	0	0	0	0	0	0	0	0	0
k__Bacteria;p__Firmicutes;c__Clostridia;o__Clostridiales;f__Lachnospiraceae;g__Lachnoclostridium;s__sp32351	0	0	0.000966744	0	0	0	0.009630446	0.009201014	0.027097385	0	0	0
k__Bacteria;p__Firmicutes;c__Clostridia;o__Clostridiales;f__Lachnospiraceae;g__Lachnoclostridium;s__sp32362-sp33711	0	0	0	0.000709287	0	0	0	0	0	0	0	0
k__Bacteria;p__Firmicutes;c__Clostridia;o__Clostridiales;f__Lachnospiraceae;g__Lachnoclostridium;s__sp32387	0	0.000795461	0	0	0	0	0.000754192	0	0	0	0.001070019	0.001429739
k__Bacteria;p__Firmicutes;c__Clostridia;o__Clostridiales;f__Lachnospiraceae;g__Lachnoclostridium;s__sp32402	0	0	0	0	0	0	0	0	0	0	0	0.005174292
k__Bacteria;p__Firmicutes;c__Clostridia;o__Clostridiales;f__Lachnospiraceae;g__Lachnoclostridium;s__sp32442	0.008478859	0.004348518	0.006831658	0	0.010762975	0.00619353	0.013807507	0.008262135	0.017453556	0.017941015	0.07363071	0.052015251
k__Bacteria;p__Firmicutes;c__Clostridia;o__Clostridiales;f__Lachnospiraceae;g__Marvinbryantia;s__sp32979	0.000786119	0	0	0	0.003348481	0.003133197	0.013575448	0.012768754	0.028753994	0	0.001538153	0.009872004
k__Bacteria;p__Firmicutes;c__Clostridia;o__Clostridiales;f__Lachnospiraceae;g__NA;s__sp32146	0.000617665	0	0	0	0	0	0	0	0	0	0	0
k__Bacteria;p__Firmicutes;c__Clostridia;o__Clostridiales;f__Lachnospiraceae;g__NA;s__sp32166	0	0	0	0	0	0	0	0	0	0	0.001136896	0
k__Bacteria;p__Firmicutes;c__Clostridia;o__Clostridiales;f__Lachnospiraceae;g__NA;s__sp32254	0.002189904	0	0	0	0	0.005173419	0	0	0	0.001777176	0.005617602	0.004833878
k__Bacteria;p__Firmicutes;c__Clostridia;o__Clostridiales;f__Lachnospiraceae;g__NA;s__sp32255	0	0	0	0	0	0	0	0	0.001301621	0	0	0
k__Bacteria;p__Firmicutes;c__Clostridia;o__Clostridiales;f__Lachnospiraceae;g__NA;s__sp32256	0	0	0	0	0	0	0.001972501	0.004976059	0.003668205	0	0	0
k__Bacteria;p__Firmicutes;c__Clostridia;o__Clostridiales;f__Lachnospiraceae;g__NA;s__sp32261	0.015217025	0	0	0	0	0	0	0	0	0	0	0
k__Bacteria;p__Firmicutes;c__Clostridia;o__Clostridiales;f__Lachnospiraceae;g__NA;s__sp32261-sp32270	0.006682015	0	0	0	0	0	0	0	0	0	0	0
k__Bacteria;p__Firmicutes;c__Clostridia;o__Clostridiales;f__Lachnospiraceae;g__NA;s__sp32270	0	0	0	0	0	0	0	0	0	0	0.002273791	0.000748911
k__Bacteria;p__Firmicutes;c__Clostridia;o__Clostridiales;f__Lachnospiraceae;g__NA;s__sp32498	0	0	0	0	0	0	0.000986251	0.001596094	0.00360904	0.000550078	0.002073163	0
k__Bacteria;p__Firmicutes;c__Clostridia;o__Clostridiales;f__Lachnospiraceae;g__NA;s__sp32510	0	0	0	0	0	0	0	0	0.001360786	0	0.001203772	0
k__Bacteria;p__Firmicutes;c__Clostridia;o__Clostridiales;f__Lachnospiraceae;g__NA;s__sp32614-sp32671	0	0	0	0	0	0	0	0	0	0	0.001805658	0.001565904
k__Bacteria;p__Firmicutes;c__Clostridia;o__Clostridiales;f__Lachnospiraceae;g__NA;s__sp32617-sp32782	0.001123028	0	0.001031194	0	0	0	0	0	0	0	0	0
k__Bacteria;p__Firmicutes;c__Clostridia;o__Clostridiales;f__Lachnospiraceae;g__NA;s__sp32622	0	0	0	0	0	0	0	0	0	0	0	0.001974401
k__Bacteria;p__Firmicutes;c__Clostridia;o__Clostridiales;f__Lachnospiraceae;g__NA;s__sp32628-sp32767	0	0	0	0	0	0	0	0	0	0	0	0.000885076
k__Bacteria;p__Firmicutes;c__Clostridia;o__Clostridiales;f__Lachnospiraceae;g__NA;s__sp32630	0	0	0	0	0	0	0	0	0.000828304	0	0	0
k__Bacteria;p__Firmicutes;c__Clostridia;o__Clostridiales;f__Lachnospiraceae;g__NA;s__sp32668	0	0	0	0	0	0	0	0	0	0	0.00247442	0
k__Bacteria;p__Firmicutes;c__Clostridia;o__Clostridiales;f__Lachnospiraceae;g__NA;s__sp32701	0	0	0	0	0	0	0	0	0	0	0.001270648	0.006808279
k__Bacteria;p__Firmicutes;c__Clostridia;o__Clostridiales;f__Lachnospiraceae;g__NA;s__sp32704	0	0	0	0	0	0	0	0.00178387	0.000828304	0	0	0.007489107
k__Bacteria;p__Firmicutes;c__Clostridia;o__Clostridiales;f__Lachnospiraceae;g__NA;s__sp32706	0	0	0	0.000851144	0	0	0	0	0	0	0	0
k__Bacteria;p__Firmicutes;c__Clostridia;o__Clostridiales;f__Lachnospiraceae;g__NA;s__sp32719-sp32755	0	0	0	0	0	0	0	0	0	0	0.001805658	0
k__Bacteria;p__Firmicutes;c__Clostridia;o__Clostridiales;f__Lachnospiraceae;g__NA;s__sp32761	0	0	0	0.005768867	0	0	0	0	0	0	0	0.000816993
k__Bacteria;p__Firmicutes;c__Clostridia;o__Clostridiales;f__Lachnospiraceae;g__NA;s__sp32763	0	0.000901522	0	0	0.00125568	0	0	0	0	0	0.004012573	0.002859477
k__Bacteria;p__Firmicutes;c__Clostridia;o__Clostridiales;f__Lachnospiraceae;g__NA;s__sp32766	0	0	0	0	0	0	0	0.001314431	0	0	0	0.006467865
k__Bacteria;p__Firmicutes;c__Clostridia;o__Clostridiales;f__Lachnospiraceae;g__NA;s__sp32778	0.001010725	0	0	0.003215434	0.000717532	0	0	0	0.000650811	0	0	0
k__Bacteria;p__Firmicutes;c__Clostridia;o__Clostridiales;f__Lachnospiraceae;g__NA;s__sp32790	0.001347633	0	0	0	0	0	0	0	0	0	0	0
k__Bacteria;p__Firmicutes;c__Clostridia;o__Clostridiales;f__Lachnospiraceae;g__NA;s__sp32791	0	0	0	0	0	0	0	0	0	0	0	0.001429739
k__Bacteria;p__Firmicutes;c__Clostridia;o__Clostridiales;f__Lachnospiraceae;g__NA;s__sp32816	0.000729968	0	0.000837845	0	0	0.001675896	0	0	0	0	0	0.002110566
k__Bacteria;p__Firmicutes;c__Clostridia;o__Clostridiales;f__Lachnospiraceae;g__NA;s__sp32819	0	0	0	0	0	0	0	0	0	0	0.001203772	0
k__Bacteria;p__Firmicutes;c__Clostridia;o__Clostridiales;f__Lachnospiraceae;g__NA;s__sp32843	0	0	0	0	0	0	0	0	0	0	0.002073163	0
k__Bacteria;p__Firmicutes;c__Clostridia;o__Clostridiales;f__Lachnospiraceae;g__NA;s__sp32856-sp32859	0	0	0	0	0	0	0	0	0	0	0	0.000953159
k__Bacteria;p__Firmicutes;c__Clostridia;o__Clostridiales;f__Lachnospiraceae;g__NA;s__sp32862	0.004716716	0.001856075	0.015790152	0.005863439	0.00693614	0.001384436	0.002378604	0.001126655	0.003372382	0.00114247	0.009429546	0.000885076
k__Bacteria;p__Firmicutes;c__Clostridia;o__Clostridiales;f__Lachnospiraceae;g__NA;s__sp32872	0.023415127	0	0	0	0	0.004371903	0	0	0	0	0.005951983	0.000953159
k__Bacteria;p__Firmicutes;c__Clostridia;o__Clostridiales;f__Lachnospiraceae;g__NA;s__sp32883	0.017350778	0.01203797	0.009280742	0	0.016204257	0.009982512	0.003074781	0.002347197	0.006212283	0	0	0
k__Bacteria;p__Firmicutes;c__Clostridia;o__Clostridiales;f__Lachnospiraceae;g__NA;s__sp32885	0.000954574	0	0	0	0	0	0	0	0	0	0.004280078	0
k__Bacteria;p__Firmicutes;c__Clostridia;o__Clostridiales;f__Lachnospiraceae;g__NA;s__sp33413	0	0	0.001997938	0	0	0.002477412	0	0	0	0	0	0
k__Bacteria;p__Firmicutes;c__Clostridia;o__Clostridiales;f__Lachnospiraceae;g__NA;s__sp33416-sp33419	0.002526812	0	0	0	0	0	0.002378604	0.002534973	0.000650811	0	0	0.001293573
k__Bacteria;p__Firmicutes;c__Clostridia;o__Clostridiales;f__Lachnospiraceae;g__NA;s__sp33419	0.000617665	0	0	0	0	0	0	0	0	0	0	0
k__Bacteria;p__Firmicutes;c__Clostridia;o__Clostridiales;f__Lachnospiraceae;g__NA;s__sp33426	0	0	0	0	0	0	0.001276324	0	0	0	0.001203772	0.000953159
k__Bacteria;p__Firmicutes;c__Clostridia;o__Clostridiales;f__Lachnospiraceae;g__NA;s__sp33432-sp33629	0.000617665	0	0	0	0	0	0	0	0	0	0	0
k__Bacteria;p__Firmicutes;c__Clostridia;o__Clostridiales;f__Lachnospiraceae;g__NA;s__sp33436	0	0	0	0	0	0	0	0	0	0	0.000735638	0
k__Bacteria;p__Firmicutes;c__Clostridia;o__Clostridiales;f__Lachnospiraceae;g__NA;s__sp33438	0	0	0	0	0	0.001967356	0	0	0	0	0	0
k__Bacteria;p__Firmicutes;c__Clostridia;o__Clostridiales;f__Lachnospiraceae;g__NA;s__sp33453	0.000954574	0	0	0	0	0	0	0	0	0	0	0
k__Bacteria;p__Firmicutes;c__Clostridia;o__Clostridiales;f__Lachnospiraceae;g__NA;s__sp33459	0	0	0	0	0	0	0	0	0	0	0	0.000816993
k__Bacteria;p__Firmicutes;c__Clostridia;o__Clostridiales;f__Lachnospiraceae;g__NA;s__sp33470	0.006625863	0.00148486	0	0	0.001315475	0.004299038	0.006265591	0.003943292	0.009466335	0.003427411	0.011368956	0.00708061
k__Bacteria;p__Firmicutes;c__Clostridia;o__Clostridiales;f__Lachnospiraceae;g__NA;s__sp33470-sp33680	0	0	0.001095643	0	0.001136092	0	0	0	0.002839901	0	0	0
k__Bacteria;p__Firmicutes;c__Clostridia;o__Clostridiales;f__Lachnospiraceae;g__NA;s__sp33503	0	0	0	0	0	0.001603031	0.001392354	0.00178387	0	0	0	0.003676471
k__Bacteria;p__Firmicutes;c__Clostridia;o__Clostridiales;f__Lachnospiraceae;g__NA;s__sp33513	0	0	0	0	0	0	0	0	0	0	0.001805658	0
k__Bacteria;p__Firmicutes;c__Clostridia;o__Clostridiales;f__Lachnospiraceae;g__NA;s__sp33518	0.008141951	0.00222729	0.026617685	0.008416872	0.002989715	0.005902069	0	0.001596094	0.001656609	0.001734862	0.007022002	0.001497821
k__Bacteria;p__Firmicutes;c__Clostridia;o__Clostridiales;f__Lachnospiraceae;g__NA;s__sp33529	0	0	0	0	0	0	0.000928236	0.001314431	0.005028991	0	0	0
k__Bacteria;p__Firmicutes;c__Clostridia;o__Clostridiales;f__Lachnospiraceae;g__NA;s__sp33539	0	0	0	0	0	0	0	0	0	0	0.001136896	0.001429739
k__Bacteria;p__Firmicutes;c__Clostridia;o__Clostridiales;f__Lachnospiraceae;g__NA;s__sp33540	0	0	0	0	0	0	0	0	0.001242457	0	0	0
k__Bacteria;p__Firmicutes;c__Clostridia;o__Clostridiales;f__Lachnospiraceae;g__NA;s__sp33558	0.002751418	0	0	0	0	0	0	0	0	0.001438666	0.005483849	0
k__Bacteria;p__Firmicutes;c__Clostridia;o__Clostridiales;f__Lachnospiraceae;g__NA;s__sp33565	0.001010725	0	0	0	0	0.003861848	0	0	0.005620637	0	0	0
k__Bacteria;p__Firmicutes;c__Clostridia;o__Clostridiales;f__Lachnospiraceae;g__NA;s__sp33582	0.00162839	0.001325768	0.002513534	0	0.001494858	0.008160886	0.004061032	0.003755516	0.003313217	0.002538823	0.006286364	0.002110566
k__Bacteria;p__Firmicutes;c__Clostridia;o__Clostridiales;f__Lachnospiraceae;g__NA;s__sp33589	0	0	0	0	0	0	0	0	0	0	0.001270648	0
k__Bacteria;p__Firmicutes;c__Clostridia;o__Clostridiales;f__Lachnospiraceae;g__NA;s__sp33597	0.000617665	0	0	0	0	0	0	0	0	0	0	0
k__Bacteria;p__Firmicutes;c__Clostridia;o__Clostridiales;f__Lachnospiraceae;g__NA;s__sp33604	0	0	0	0	0	0	0.018100598	0.021030889	0.028635664	0	0	0
k__Bacteria;p__Firmicutes;c__Clostridia;o__Clostridiales;f__Lachnospiraceae;g__NA;s__sp33616-sp33639	0.011398731	0.001166676	0.00328693	0.000851144	0.004066013	0.003570388	0.00539537	0.007041592	0.022600876	0.002581137	0	0.000953159
k__Bacteria;p__Firmicutes;c__Clostridia;o__Clostridiales;f__Lachnospiraceae;g__NA;s__sp33636	0.001852996	0	0	0	0	0	0	0	0	0.000592392	0.01324149	0.00292756
k__Bacteria;p__Firmicutes;c__Clostridia;o__Clostridiales;f__Lachnospiraceae;g__NA;s__sp33639	0.00364984	0	0	0	0	0	0.010210593	0.006008825	0.009407171	0	0.021199759	0.003472222
k__Bacteria;p__Firmicutes;c__Clostridia;o__Clostridiales;f__Lachnospiraceae;g__NA;s__sp33658	0.001403785	0	0	0	0	0.001821626	0.001624413	0.001502206	0.00295823	0	0	0.001089325
k__Bacteria;p__Firmicutes;c__Clostridia;o__Clostridiales;f__Lachnospiraceae;g__NA;s__sp33670	0.000673817	0	0	0	0.00125568	0	0.002610663	0.002628861	0.001183292	0	0.001070019	0.001497821
k__Bacteria;p__Firmicutes;c__Clostridia;o__Clostridiales;f__Lachnospiraceae;g__NA;s__sp33674	0	0.005833378	0.00489817	0.004397579	0.006756757	0.01296998	0.006671695	0.009294902	0.007395574	0	0	0
k__Bacteria;p__Firmicutes;c__Clostridia;o__Clostridiales;f__Lachnospiraceae;g__NA;s__sp33694	0.007243529	0.003553057	0.004124774	0	0.008012437	0.007796561	0	0	0	0.000507765	0.002340667	0
k__Bacteria;p__Firmicutes;c__Clostridia;o__Clostridiales;f__Lachnospiraceae;g__NA;s__sp33734	0	0	0	0	0	0.001384436	0	0	0	0.000677019	0	0
k__Bacteria;p__Firmicutes;c__Clostridia;o__Clostridiales;f__Lachnospiraceae;g__NA;s__sp33738	0.002807569	0	0.003802526	0	0	0.002841737	0	0.001314431	0.008283043	0.002157999	0.003678192	0
k__Bacteria;p__Firmicutes;c__Clostridia;o__Clostridiales;f__Lachnospiraceae;g__NA;s__sp33760	0	0	0	0	0	0	0	0.001032767	0	0	0.000936267	0
k__Bacteria;p__Firmicutes;c__Clostridia;o__Clostridiales;f__Lachnospiraceae;g__NA;s__sp33761	0.000673817	0	0	0	0	0.001457301	0	0	0.001479115	0	0.006955126	0
k__Bacteria;p__Firmicutes;c__Clostridia;o__Clostridiales;f__Lachnospiraceae;g__NA;s__sp33766	0	0	0	0	0	0	0.001276324	0.001971646	0	0	0.002273791	0.000748911
k__Bacteria;p__Firmicutes;c__Clostridia;o__Clostridiales;f__Lachnospiraceae;g__NA;s__sp33767	0	0	0	0	0	0	0	0	0	0	0.002541296	0.000748911
k__Bacteria;p__Firmicutes;c__Clostridia;o__Clostridiales;f__Lachnospiraceae;g__NA;s__sp33781	0	0	0.001224542	0	0	0	0	0	0	0	0	0
k__Bacteria;p__Firmicutes;c__Clostridia;o__Clostridiales;f__Lachnospiraceae;g__Roseburia;s__sp33136	0	0	0	0	0	0	0.000638162	0	0.00076914	0	0	0
k__Bacteria;p__Firmicutes;c__Clostridia;o__Clostridiales;f__Lachnospiraceae;g__Roseburia;s__sp33136-sp33149	0	0	0	0	0	0	0	0	0	0	0.000735638	0
k__Bacteria;p__Firmicutes;c__Clostridia;o__Clostridiales;f__Lachnospiraceae;g__Roseburia;s__sp33140	0	0	0	0	0	0	0	0	0.00076914	0	0	0
k__Bacteria;p__Firmicutes;c__Clostridia;o__Clostridiales;f__Lachnospiraceae;g__Roseburia;s__sp33143	0	0	0	0	0	0	0.001914486	0.004318843	0.002780736	0	0	0
k__Bacteria;p__Firmicutes;c__Clostridia;o__Clostridiales;f__Lachnospiraceae;g__Roseburia;s__sp33149	0	0	0	0	0	0	0	0	0	0.000846274	0.001805658	0
k__Bacteria;p__Firmicutes;c__Clostridia;o__Clostridiales;f__Lachnospiraceae;g__Roseburia;s__sp33152	0.001740693	0.002015167	0.001353442	0	0.006637168	0.001967356	0	0	0	0	0	0.00245098
k__Bacteria;p__Firmicutes;c__Clostridia;o__Clostridiales;f__Lachnospiraceae;g__Roseburia;s__sp33168	0	0	0	0	0	0	0	0.001220543	0	0	0.003544439	0
k__Bacteria;p__Firmicutes;c__Clostridia;o__Clostridiales;f__Lachnospiraceae;g__Roseburia;s__sp33177	0	0	0	0	0	0	0	0	0	0	0.000735638	0
k__Bacteria;p__Firmicutes;c__Clostridia;o__Clostridiales;f__NA;g__*Arthromitus;s__sp30492	0	0	0	0	0	0	0	0	0.008046385	0	0	0
k__Bacteria;p__Firmicutes;c__Clostridia;o__Clostridiales;f__Peptostreptococcaceae;g__Terrisporobacter;s__sp34393	0	0	0	0	0	0	0	0	0	0	0	0.000748911
k__Bacteria;p__Firmicutes;c__Clostridia;o__Clostridiales;f__Ruminococcaceae;g__NA;s__sp34731	0	0	0	0	0.001016503	0	0	0	0	0	0	0
k__Bacteria;p__Firmicutes;c__Clostridia;o__Clostridiales;f__Ruminococcaceae;g__NA;s__sp34764	0	0	0	0	0	0.001238706	0.000812206	0	0	0	0	0
k__Bacteria;p__Firmicutes;c__Clostridia;o__Clostridiales;f__Ruminococcaceae;g__NA;s__sp34771	0	0	0	0	0	0	0.001044265	0.001032767	0.005265649	0	0	0
k__Bacteria;p__Firmicutes;c__Clostridia;o__Clostridiales;f__Ruminococcaceae;g__NA;s__sp34795-sp34820	0.001291482	0	0	0	0.000717532	0.001603031	0	0	0.004200686	0	0	0
k__Bacteria;p__Firmicutes;c__Clostridia;o__Clostridiales;f__Ruminococcaceae;g__NA;s__sp35297	0	0	0	0	0	0	0.002726693	0.001126655	0.001479115	0	0.00581823	0
k__Bacteria;p__Firmicutes;c__Clostridia;o__Clostridiales;f__Ruminococcaceae;g__NA;s__sp35310	0	0	0.000837845	0	0.001375269	0.001238706	0	0	0	0	0	0
k__Bacteria;p__Firmicutes;c__Clostridia;o__Clostridiales;f__Ruminococcaceae;g__NA;s__sp35328-sp35341-sp35377	0	0	0	0	0	0	0	0	0	0	0.002407544	0
k__Bacteria;p__Firmicutes;c__Clostridia;o__Clostridiales;f__Ruminococcaceae;g__NA;s__sp35341	0	0	0	0	0	0	0	0	0	0	0.001671905	0.003063725
k__Bacteria;p__Firmicutes;c__Clostridia;o__Clostridiales;f__Ruminococcaceae;g__NA;s__sp35351	0	0	0	0	0	0	0	0	0	0	0.00300943	0
k__Bacteria;p__Firmicutes;c__Clostridia;o__Clostridiales;f__Ruminococcaceae;g__NA;s__sp35360	0.000842271	0	0.000708946	0	0	0	0	0	0	0	0	0
k__Bacteria;p__Firmicutes;c__Clostridia;o__Clostridiales;f__Ruminococcaceae;g__NA;s__sp35373	0	0	0.002642434	0	0	0	0	0	0	0	0	0
k__Bacteria;p__Firmicutes;c__Clostridia;o__Clostridiales;f__Ruminococcaceae;g__NA;s__sp35380	0	0	0.001031194	0	0	0	0	0	0	0	0	0
k__Bacteria;p__Firmicutes;c__Clostridia;o__Clostridiales;f__Ruminococcaceae;g__NA;s__sp35382	0	0	0	0	0	0	0	0	0	0	0.005951983	0.001429739
k__Bacteria;p__Firmicutes;c__Clostridia;o__Clostridiales;f__Ruminococcaceae;g__NA;s__sp35382-sp35403	0.00769274	0	0	0	0	0	0	0	0	0	0	0
k__Bacteria;p__Firmicutes;c__Clostridia;o__Clostridiales;f__Ruminococcaceae;g__NA;s__sp35382-sp35403-sp35432	0	0	0	0	0	0	0	0	0	0	0.002808801	0
k__Bacteria;p__Firmicutes;c__Clostridia;o__Clostridiales;f__Ruminococcaceae;g__NA;s__sp35403	0.002246055	0.000689399	0	0	0.001016503	0	0.001624413	0.001032767	0.002721571	0	0	0
k__Bacteria;p__Firmicutes;c__Clostridia;o__Clostridiales;f__Ruminococcaceae;g__NA;s__sp35424	0	0	0.001869038	0	0.000777326	0.002040222	0	0	0	0.001607921	0.009496422	0
k__Bacteria;p__Firmicutes;c__Clostridia;o__Clostridiales;f__Ruminococcaceae;g__NA;s__sp35432	0.001179179	0	0	0	0	0	0	0	0.00141995	0	0	0
k__Bacteria;p__Firmicutes;c__Clostridia;o__Clostridiales;f__Ruminococcaceae;g__NA;s__sp35498	0	0	0	0	0.001674241	0.002040222	0	0	0.00076914	0	0.000735638	0
k__Bacteria;p__Firmicutes;c__Clostridia;o__Clostridiales;f__Ruminococcaceae;g__NA;s__sp35597-sp35598	0	0	0	0	0	0	0	0	0	0	0.001671905	0
k__Bacteria;p__Firmicutes;c__Clostridia;o__Clostridiales;f__Ruminococcaceae;g__NA;s__sp35598	0.001179179	0	0.001804589	0	0.000777326	0	0.001682427	0.001877758	0.002366584	0	0	0
k__Bacteria;p__Firmicutes;c__Clostridia;o__Clostridiales;f__Ruminococcaceae;g__NA;s__sp35736	0.000617665	0	0	0	0.000717532	0	0.000638162	0.001126655	0.002425748	0.000592392	0.002006286	0.001021242
k__Bacteria;p__Firmicutes;c__Clostridia;o__Clostridiales;f__Ruminococcaceae;g__NA;s__sp35799	0	0	0	0	0	0	0	0	0	0	0	0.001293573
k__Bacteria;p__Firmicutes;c__Clostridia;o__Clostridiales;f__Ruminococcaceae;g__NA;s__sp35832-sp35839	0	0	0	0	0	0	0	0	0	0	0.001337524	0
k__Bacteria;p__Firmicutes;c__Clostridia;o__Clostridiales;f__Ruminococcaceae;g__Oscillibacter;s__sp34650-sp34654	0	0	0	0	0	0	0	0	0	0	0.000802515	0
k__Bacteria;p__Firmicutes;c__Clostridia;o__Clostridiales;f__Ruminococcaceae;g__Ruminiclostridium;s__sp34857-sp34950	0	0	0	0	0.001016503	0	0	0	0	0	0	0
k__Bacteria;p__Firmicutes;c__Clostridia;o__Clostridiales;f__Ruminococcaceae;g__Ruminiclostridium;s__sp34925	0.000729968	0	0	0	0	0	0.002900737	0.003286076	0	0	0	0.000748911
k__Bacteria;p__Firmicutes;c__Clostridia;o__Clostridiales;f__Ruminococcaceae;g__Ruminococcus;s__bromii	0	0	0	0	0	0	0	0	0	0	0.001605029	0
k__Bacteria;p__Firmicutes;c__Erysipelotrichia;o__Erysipelotrichales;f__Erysipelotrichaceae;g__Allobaculum;s__sp36555	0	0	0	0	0	0	0	0	0	0.000846274	0	0.003267974
k__Bacteria;p__Firmicutes;c__Erysipelotrichia;o__Erysipelotrichales;f__Erysipelotrichaceae;g__Erysipelatoclostridium;s__sp36616-sp36629	0	0.000583338	0	0	0	0	0.005627429	0.005914938	0.003313217	0	0	0.002042484
k__Bacteria;p__Firmicutes;c__Erysipelotrichia;o__Erysipelotrichales;f__Erysipelotrichaceae;g__NA;s__sp36783	0	0	0	0	0	0	0.001334339	0	0.000828304	0	0.001136896	0.001770153
k__Bacteria;p__Firmicutes;c__Erysipelotrichia;o__Erysipelotrichales;f__Erysipelotrichaceae;g__NA;s__sp36786	0	0	0	0	0	0	0.00220456	0.00225331	0.00076914	0	0.000802515	0
k__Bacteria;p__Firmicutes;c__Erysipelotrichia;o__Erysipelotrichales;f__Erysipelotrichaceae;g__NA;s__sp36793	0	0	0	0	0	0	0.001160295	0	0	0	0	0
k__Bacteria;p__Firmicutes;c__Erysipelotrichia;o__Erysipelotrichales;f__Erysipelotrichaceae;g__Turicibacter;s__sanguinis	0.017182324	0.008590974	0.012245424	0.014564025	0.020928008	0.002550277	0	0	0	0	0	0
k__Bacteria;p__Proteobacteria;c__Alphaproteobacteria;o__Rhizobiales;f__Methylocystaceae;g__Methylocystis;s__Methylocystis-echinoides-parvus	0	0	0	0	0	0	0	0	0	0	0	0.000885076
k__Bacteria;p__Proteobacteria;c__Alphaproteobacteria;o__Rhodobacterales;f__Rhodobacteraceae;g__Paracoccus;s__sp44877	0	0	0	0	0	0	0	0	0	0.000634706	0	0.00170207
k__Bacteria;p__Proteobacteria;c__Alphaproteobacteria;o__Rickettsiales;f__Mitochondria;g__Arundo-Oryza meyeriana;s__NA	0	0	0	0	0	0	0	0	0	0	0	0.000748911
k__Bacteria;p__Proteobacteria;c__Betaproteobacteria;o__Burkholderiales;f__Alcaligenaceae;g__Parasutterella;s__excrementihominis	0.001572239	0.00450761	0.004962619	0.004350293	0.00263095	0.001967356	0	0	0	0	0	0
k__Bacteria;p__Tenericutes;c__Mollicutes;o__Anaeroplasmatales;f__Anaeroplasmataceae;g__Anaeroplasma;s__sp67615	0	0	0	0	0	0	0	0	0	0	0	0.000953159
k__Bacteria;p__Tenericutes;c__Mollicutes;o__NA;f__NA;g__NA;s__sp67846	0	0	0	0	0	0	0	0	0	0	0.000936267	0
k__Bacteria;p__Verrucomicrobia;c__Verrucomicrobiae;o__Verrucomicrobiales;f__Verrucomicrobiaceae;g__Akkermansia;s__muciniphila	0.014262452	0.024235032	0.037574117	0.004917723	0.036893088	0.014864471	0.031560016	0.021781992	0.003194888	0	0	0
None;Other;Other;Other;Other;Other;Other	0	0	0	0	0	0.001384436	0	0.001126655	0	0.000465451	0	0
