# Supplementary material for: Gut lumen-leaked microbial DNA causes myocardial inflammation and impairs cardiac contractility in ageing mouse heart
Source: Front Immunol. 2023 Jul 13;14:1216344. doi: 10.3389/fimmu.2023.1216344 (PMC10373503; doi:10.3389/fimmu.2023.1216344)
Supplement: Supplementary file 4 [file Table_3.pdf]

Table S3. Human sample information			
**ID**	**Source**	**Age**	**Gender**
T2234122	BioChain	36	Male
T2234126	BioChain	30	Male
T2234127	BioChain	36	Male
T2234132	BioChain	68	Female
T2234135	BioChain	77	Male
T2236122Hd-2	BioChain	82	Female
